# Modular Point-of-Need Tropane Alkaloid Detection at Regulatory Levels: Combining Solid–Liquid Extraction from Buckwheat with a Paper-Immobilized Liquid-Phase Microextraction and Immuno-Detection in Interconnectable 3D-Printed Devices

**DOI:** 10.1021/acs.analchem.4c04811

**Published:** 2024-10-04

**Authors:** Ids B. Lemmink, Linda Willemsen, Erik Beij, Toine F. H. Bovee, Han Zuilhof, Gert IJ. Salentijn

**Affiliations:** † Laboratory of Organic Chemistry, Wageningen University & Research, Stippeneng 4, 6708 WE, Wageningen, The Netherlands; ‡ Wageningen Food Safety Research, Wageningen University & Research, Akkermaalsbos 2, 6708 WB Wageningen, The Netherlands; § School of Pharmaceutical Sciences and Technology, Tianjin University, 92 Weijin Road, Tianjin, 300072, China

## Abstract

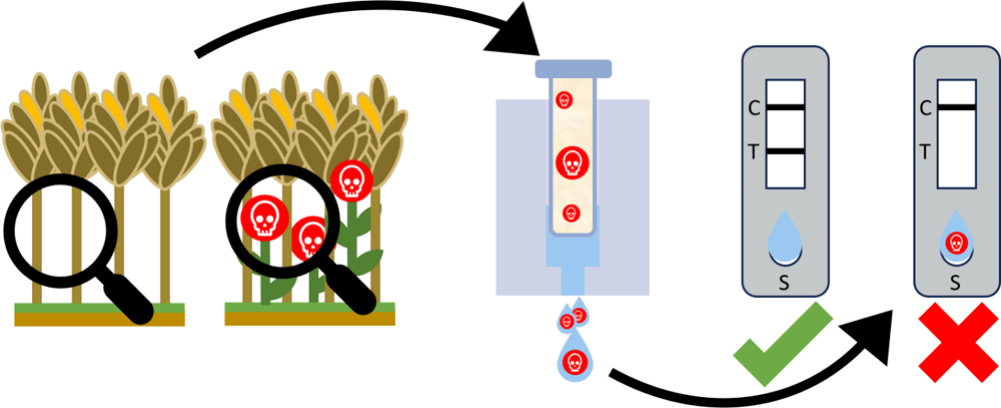

Contamination with tropane alkaloids in cereals is expected to increase globally. However, current identification tools (e.g., liquid chromatography–mass spectrometry) for tropane alkaloids are time-consuming and expensive. Furthermore, their miniaturized alternatives lack sensitivity and robustness. Therefore, there is a pressing need for inexpensive and effective screening methods. Here, an on-site applicable modular workflow for tropane alkaloid detection in buckwheat is presented. The modular workflow combines paper microfluidics and interconnectable 3D-printed sample preparation tools and was evaluated for different tropane alkaloids, including atropine and scopolamine. Furthermore, integration with an indirect competitive lateral flow immunoassay (icLFIA) for atropine detection at relevant levels was demonstrated. In the modular workflow, to minimize matrix coextraction, tropane alkaloids were extracted from the milled buckwheat cereals by a mixture of alkaline aqueous and immiscible organic solvents (extraction recoveries: 66–79%). The tropane alkaloids were subsequently concentrated with a newly developed paper-immobilized liquid-phase microextraction (PI-LPME, extraction recoveries: 34–60%, concentration factor to immobilized solution in paper: 60–108×). After the PI-LPME, with an integrated 3D-printed setup, the tropane alkaloids were directly eluted (elution recoveries: 83–93%) and detected with the icLFIA. Digital read-out of the icLFIA, by employing a hand-held reader, enabled semiquantification of atropine (IC_50_ = 0.56 ng mL^–1^ in standard solutions). The modular workflow was validated by analyzing 24 blank and spiked buckwheat cereal samples with 5 and 10 μg kg^–1^ atropine. A cutoff value was established with an estimated false negative rate of 1% and estimated false positive rate of 0.68%. Therefore, the modular workflow can aid in fast, inexpensive, and on-site atropine detection by nonexperts, and when integrated with a scopolamine-specific icLFIA expanded toward scopolamine detection. Moreover, the developed sample extraction and concentration method (PI-LPME) is suitable for the analysis of many other compounds with pH-dependent polarity.

## Introduction

Tropane alkaloids are hazardous secondary plant metabolites mainly produced by invasive plants that easily grow between crops of different food commodities, such as cereal and soy.^1^ Therefore, during harvest, cross-crop contamination occurs frequently. Between 2005 and 2022, 80 incidents with tropane alkaloid-contaminated food products within the European Union (EU) have been reported.^2^ Climate change, increasing eco-production, and the rise of herbicide-resistant weeds will make this number almost inevitably increase.^3^ The product categories with the greatest number of contamination incidents were buckwheat, corn-based food products, and millet-based food.^2^ From the 200 tropane alkaloids described in the literature, the two occurring most frequently are atropine and scopolamine. On account of their regular occurrence and adverse health effects, atropine and scopolamine are regulated in the EU.^2^ The maximum level for the sum of atropine and scopolamine in milled buckwheat cereals and products has been set at 10 μg kg^–1^ (Commission Regulation (EU) 2023/915, on maximum levels for certain contaminants in food). Although regulated as the sum of atropine and scopolamine, atropine is the most prominent tropane alkaloid in buckwheat cereals, as looking at all reported buckwheat cereal contamination cases with tropane alkaloids in the EU in the last 15 years, atropine was always the tropane alkaloid present at the highest concentration (14–240 μg kg^–1^, RASFF database).^2^ Other tropane alkaloids that have been reported in food within the EU are homatropine, anisodine, aposcopolamine, and anisodamine.^2^ Although not legislated, these tropane alkaloids can have an adverse effect on the peripheral nervous system.^1^ For more details about the structure, molecular weight, predicted log(*P*) in a 1-octanol/water system, and p*K*_a_ values of these tropane alkaloids, see Supporting Information (SI), Figure S1.

At present, the analysis of tropane alkaloids is mainly dependent on instrumental methods, such as liquid chromatography–mass spectrometry (LC-MS) or gas chromatography–mass spectrometry (GC-MS).^4^ The limit of detection (LOD) obtained with these hyphenated techniques is roughly between 0.05 and 2 μg kg^–1^. Although such methods for alkaloid detection are the gold standard, they are expensive.^5^ Immunochemical screening assays, such as enzyme-linked immunosorbent assays or microsphere-based immunoassays, can be alternatives.^6^ However, these techniques still require advanced laboratory equipment, trained personnel, and extensive manual labor.^3^ Therefore, these techniques are restricted to tests in centralized laboratories. There is, thus, a pressing need to develop innovative sensing technologies that allow inexpensive and fast on-site screening of tropane alkaloids in food commodities.^2,3^ These screening methods can be used as a preliminary test, after which the samples screened positive are sent toward a laboratory for confirmatory analysis, when possible. Following EU-regulation 2023/2783, these screening methods should be validated to be fit-for-purpose by establishing a cutoff value with a false negative rate of 5% and report the corresponding false positive rate.

Lateral flow immunoassays (LFIAs) are inexpensive immunoassays, combining antibodies as recognition elements with optical signal generation that can be interpreted visually, making it an attractive form of biosensing at the point-of-need.^7^ Many people have already become accustomed with using LFIAs, e.g., SARS-CoV-19 tests. Similar to the SARS-CoV-19 test, the LFIA for food safety applications can be used as a screening test. Poor matrix tolerance and low sensitivity are, however, main bottlenecks hindering the widespread application of LFIAs in the field of food safety.^3^ The LFIAs already developed for tropane alkaloid detection predominantly focus on (viscous) liquid matrices (e.g., saliva, urine, or honey) that do not require a solid–liquid extraction (SLE), have LODs above the European regulatory limits or require a workflow with extensive laboratory-oriented sample preparation.^8−10^ Electrochemical sensors, on the other hand, can detect tropane alkaloids in drinks, urine, and, tomatoes with varying sensitivities (LODs: 0.1–1000 ng mL^–1^).^11^ Although cereals and tomatoes are both solid food commodities, their compositions are completely different. Following EU-regulation 2023/2783, for the analysis of plant toxins in foods, buckwheat cereals fall in the category of high starch and low water content, whereas tomatoes fall in the category of high water content. The analysis methods validated for compounds in one food category cannot be used for the analysis of food commodities in another category without further validation (EU-regulation 2023/2783). Therefore, until now, no on-site applicable methods for tropane alkaloid detection at regulatory levels in milled buckwheat cereals exist.

Typically, tropane alkaloid detection protocols start with an acidic SLE.^1^ After the SLE, further matrix cleanup is often required before analysis.^4^ Quick, Easy, Cheap, Effective, Rugged and Safe (QuEChERS) protocols have been developed as a replacement of the traditional SLE, as during QuEChERS the analyte of interest is extracted from the food commodity, while at the same time the method provides matrix cleanup.^1^ Although QuEChERS protocols are considered environmentally friendly, their application potential at the point-of-need is limited, as the procedure requires multiple handling steps, and miniaturization remains a challenge.^1,12,13^ Other matrix cleanup methods include a solid-phase extraction (SPE) or liquid–liquid extraction (LLE).^1^ Currently, most SPE protocols either employ commercial strong cation exchange or molecular imprinted polymers.^14,15^ Many SPE protocols have a lab-oriented workflow.^14,15^ Conventional LLE protocols are lengthy and consume large amounts of solvents. Solid or membrane supported liquid phase microextractions (LPMEs) have been developed to overcome the amount of solvent required during a LLE.^16^ Current research in the field of LPME focuses on the development of inexpensive and biodegradable supports.^17^ Furthermore, integrated LPME protocols for application at the point-of-need are still lacking.^16^

At the same time, microfluidic sample preparation platforms have been developed for rapid sample purification and analyte concentration in resource-limited settings.^18^ Especially, paper microfluidic systems have substantial potential for point-of-need application, as the material costs are minimal and there is no need for pumps, as fluidic transport is based on capillary action.^19,20^ Previously, with paper microfluidics, a countercurrent LLE on paper has been reported, in which it has been proven that compounds can be transferred reversibly between regular and hydrophobically modified paper based on pH, as the organic and aqueous solution could only wet one of the two pieces of paper.^21^ However, bottlenecks in many current paper microfluidic systems are that they are fragile, lack robustness, and often require a high amount of user involvement during the device operation.^19^ 3D-printing technology has the potential to mitigate these drawbacks of paper microfluidic systems by the fabrication of housings for the device, enhancing reproducibility and user-friendliness.^22,23^ The accessibility of 3D-printing enables the decentralized production of point-of-need devices on-demand.^24^

The current manuscript describes the development of a modular workflow, which combines paper microfluidics and interconnectable 3D-printed sample preparation tools (evaluated for different tropane alkaloids), with an icLFIA with a digital read-out for atropine detection at relevant levels in milled buckwheat cereals.

## Materials and Methods

### Experimental Strategy

**Figure 1:**
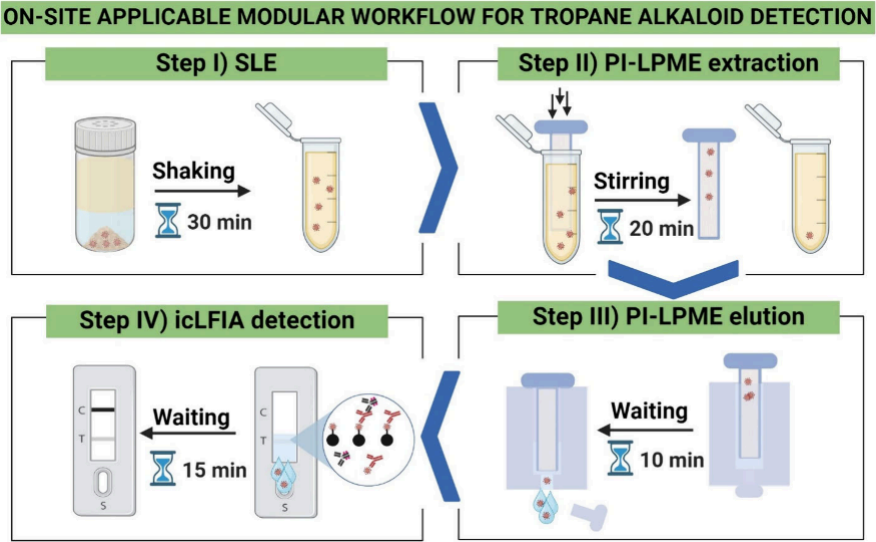
Overview of the on-site applicable modular workflow for tropane alkaloid detection in buckwheat cereals at the point-of-need (created with biorender.com): Sample preparation: Atropine is extracted from the buckwheat cereals (step I), concentrated using a newly developed paper-based LLE (step II), and subsequently, atropine is recovered from the paper (step III). Detection: The eluted atropine is directly measured with icLFIA (step IV).

The aim of this work was to develop a modular workflow for tropane alkaloid detection in milled buckwheat cereals at the point-of-need. In Figure 1, an overview of the optimized modular workflow is presented (see Figure S2 for a step-by-step protocol of the modular workflow and Figure S3 for an overview of the development and optimization). During the development of the modular workflow, an extract collector was developed, combining readily available laboratory equipment with a 3D-printed SLE-filter attachment, to ensure a rapid, safe, and user-friendly SLE (step I). The SLE-extracts were analyzed by LC-MS/MS to optimize the extraction solvent composition and extraction time. Then, for step II, a paper-immoblized liquid-phase microextraction (PI-LPME) method was developed. During the PI-LPME, tropane alkaloids dissolved in an organic solution are enriched on a small piece of cellulose paper, prewetted with immobilized acidic aqueous solution, thus allowing the partitioning of protonated bases into it. The organic solution before and after PI-LMPE was analyzed by LC-MS/MS to optimize the extraction time. A 3D-printed PI-LPME frame was developed to enhance the reproducibility and user-friendliness. Subsequently, for step III, a 3D-printed PI-LPME holder was developed, enabling the elution of the tropane alkaloids from the paper. The extracts were analyzed by LC-MS/MS to optimize the elution time and elution solvent composition. The applicability of the sample preparation of the modular workflow (steps I–III), was evaluated for atropine, scopolamine, homatropine, and anisodine by performing the complete workflow while quantifying the tropane alkaloid concentrations at all intermediate steps with LC-MS/MS. To demonstrate the applicability of the modular workflow for tropane alkaloid detection at regulatory levels with an icLFIA, for step IV, the sensitivity and specificity of the used immunoassay were first evaluated with an indirect competitive microsphere-based immunoassay. Subsequently, a specific-icLFIA for atropine was developed. After the icLFIA was developed, by employing a hand-held reader, a calibration curve with atropine in standard solutions was made. Finally, the modular workflow with an atropine-specific icLFIA was validated, following EU-regulation 2023/2783, by analyzing blank and spiked buckwheat cereal samples with 5 and 10 μg kg^–1^ atropine. The experimental details including chemicals and consumables (see SI, Protocol S1), instrument setups (see SI, Protocol S2), experimental procedures (see SI, Protocol S3), HPLC-MS/MS acquisition parameters (see SI, Table S1), HPLC-MS/MS calibration curves (see SI, Figure S4), 3D-printer settings (see SI, Table S2), and schematic overview of the icLFIA (see SI, Figure S5) can be found in the experimental section of the SI.

## Results and Discussion

### Tools for the Modular Workflow

**Figure 2:**
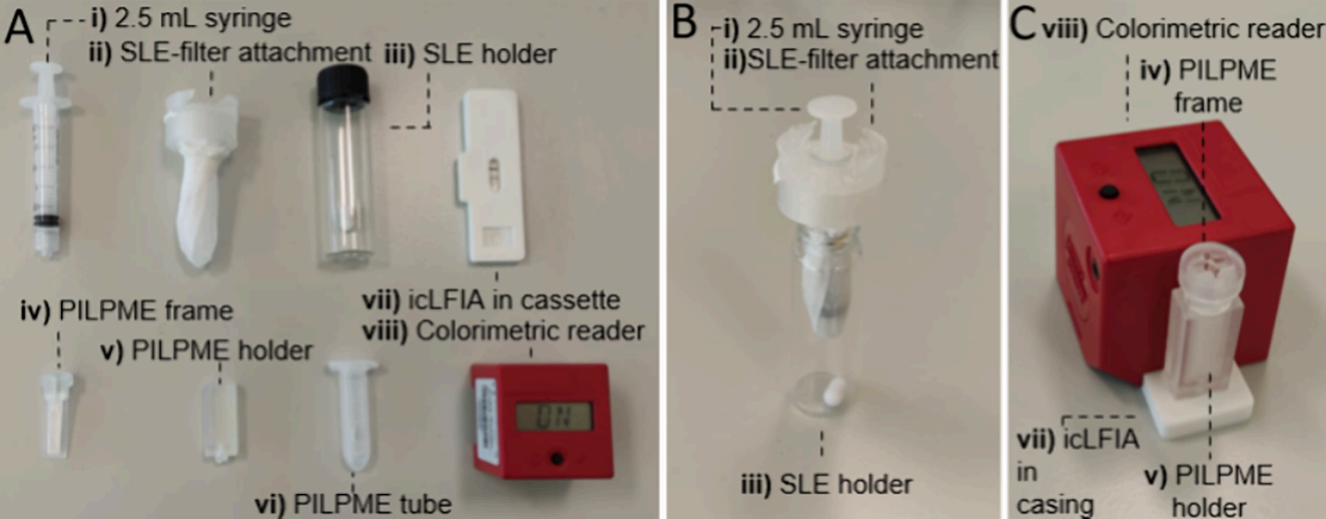
Overview of the tools required for the modular workflow. (A) All disassembled tools and their corresponding identification number (as used through the section Tools for the Modular Workflow): (i) 2.5 mL syringe, (ii) SLE-filter attachment, (iii) SLE holder, (iv) PI-LPME frame, (v) PI-LPME holder, (vi) PI-LPME tube, (vii) icLFIA in cassette, and (viii) colorimetric reader. (B) Side view of the assembled parts of the device where the SLE-filter attachment (ii) is attached to the 2.5 mL syringe (i), which is inserted in the SLE-holder (iii). (C) Side view of the assembled parts of the device where the PI-LPME frame (iv) is inserted in the PI-LPME holder (v), and the PI-LMPE holder is connected to the icLFIA in the cassette (vii). The colorimetric reader (viii) is clicked on top of the icLFIA allowing for a colorimetric read-out of the test line and control line intensity.

To enhance the application potential, the modular workflow is constructed from readily available laboratory consumables and 3D-printed devices (Figure 2A). The on-site application potential of the SLE is mainly enhanced by the developed SLE-filter attachment (ii; see SI, Figure S6 for specifications). The SLE is performed in a 16 mL glass vail containing a 10 mm × 6 mm stirring bar (iii) to prevent the aggregation of buckwheat cereals, and enhance the extraction. After SLE optimization, an extraction solvent mixture of 2 mL of 0.05 M NaOH in water and 10 mL of butyl acetate was selected (see Solid–Liquid Extraction). After the extraction, the 2.5 mL syringe (i) can be clicked into the SLE-filter attachment (ii; see Figure 2B). The tea filter in the SLE-filter attachment (ii), with an average pore size of approximately 50 μm, prevents the buckwheat cereals from entering the syringe (i) during the extract collection. After the extract is collected, the syringe (i) can be decoupled easily from the SLE-filter attachment (ii) by turning. The SLE-filter attachment (ii) stays behind, thus closing of the SLE holder (iii), which is now ready for disposal. With the SLE-filter attachment (ii), potential contact of the end-user with the extract is minimized. Moreover, the SLE-filter attachment (ii) eliminates the necessity for centrifugation steps or long waiting times for separation by gravity, as is typically encountered in other on-site applicable methods.^6,10,25^

Next, PI-LPME extraction from that extract could be performed. Water can move into paper by capillary action more readily than butyl acetate. Thus, when a small piece of paper is prewetted with acidic aqueous solution and submerged in butyl acetate, the aqueous solution is immobilized in the paper. All tropane alkaloids are weak bases with a predicted p*K*_a_ value between 7.8 and 10.2.^26^ At a high pH (pH ≫ 10.2), most tropane alkaloids are deprotonated and have the highest affinity for the apolar organic solution, whereas at a low pH (pH ≪ 7.8), most tropane alkaloids are protonated and have the highest affinity for the polar aqueous solution. Paper was prewetted with 0.1% (v/v%) formic acid in water, and subsequently submerged in the apolar organic extraction solvent containing tropane alkaloids. The tropane alkaloids present are thus concentrated on the paper, while most nonionizable matrix compounds remain in the extract.

The PI-LPME was simplified by the development of a 3D-printed PI-LPME frame (iv) and 3D-printed PI-LPME holder (v; see Figure 2A and SI, Figures S7 and S8 for specifications). The PI-LPME frame (iv) ensures a reproducible and stable positioning of the paper during the PI-LPME extraction and elution and prevents any direct contact between the paper and the end-user. The PI-LPME frame (iv) fits on the PI-LPME tube (vi), while the length of the frame still enables stirring during the PI-LPME extraction; the tube is longer than the PI-LPME frame. After the PI-LPME extraction, the PI-LPME frame (iv) can be fit in the PI-LPME holder (v). The PI-LPME holder (v) contains 200 μL of RB as an elution solvent. With the PI-LPME frame (iv) inside the PI-LPME holder (v), the spare volume is only 20 μL, ensuring proper wetting of the PI-LPME paper once inserted. At the bottom of the PI-LPME holder (v), there is a small hole that is closed with a stopper during the extraction. To elute the RB from the PI-LPME holder (v), the stopper can be removed. Due to the surface tension of the RB, the RB will not flow through the hole upon removal of the stopper. The PI-LPME holder (v) can be first clicked on top of the icLFIA cassette (vii). After this, the surface tension of the RB can be overcome by moving the SPME frame up and down to pass the RB onto the icLFIA. The volume of RB leaving the PI-LPME holder toward the icLFIA was 124 μL (SD = 7 μL, n = 5). The remainder of the RB remains in the holder and on the paper.

The icLFIA analysis is simplified by the development of a 3D-printed icLFIA cassette (vii; see Figure 2A and SI, Figure S9 for specifications). The cassette provides stability and protection for the icLFIA, during usage. After the icLFIA run is complete, the colorimetric reader (viii) can be clicked on the icLFIA cassette (vii) for a digital read-out of the test line and control line intensity.

### Development of Sample Preparation of the Modular Workflow

#### Solid–Liquid Extraction

**Figure 3:**
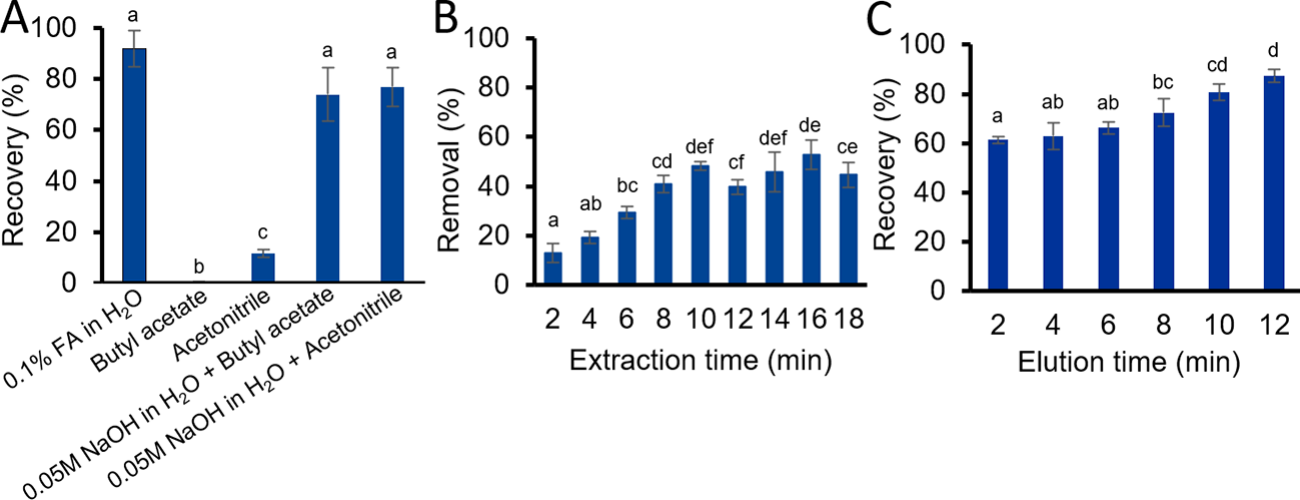
Atropine recovery (%) or removal (%) obtained with step I–III of the modular workflow for tropane alkaloid-detection in buckwheat cereals with (A) an SLE with different extraction solvents, (B) different PI-LPME extraction times (min), or (C) different PI-LPME elution times (min). For each step, the recovery (%) or removal (%) was determined with LC-MS/MS analysis. Error bars represent the standard deviation (*n* = 3). In each figure, data points that do not share a letter are significantly different, determined by one-way ANOVA, followed by Post Hoc Tukey test (*p* < 0.05).

Acidic polar (organic) extraction solvents are commonly used for the SLE of tropane alkaloids from cereals and cereal-based products, as under acidic conditions tropane alkaloids are protonated and therefore, highly soluble in water.^4,27^ For this reason, atropine was first extracted from the buckwheat cereals using 0.1% (v/v%) formic acid in water, resulting in an atropine recovery of approximately 90% (Figure 3A). However, despite filtration, the extract was viscous and cloudy, indicating that a lot of matrix was coextracted (see SI, Figure S10A). When blank acidic cereal extracts were diluted with running buffer, only after a 10× dilution with RB, the T-line signal of the icLFIA was similar to a blank signal in clean RB (see SI, Figure S11). Therefore, a significant dilution before icLFIA analysis is required, which inhibits atropine detection at low concentrations. Similar dilution steps were reported, when instead of 0.1% (v/v%) formic acid in water, 0.1% (v/v%) formic acid in a mixture of methanol and water is used.^10^

In order to minimize matrix coextraction, atropine was extracted from the buckwheat cereals using (a combination of alkaline aqueous and) organic solvents. With acetonitrile (ACN) and butyl acetate (BA) as extraction solvents, matrix coextraction was minimized (see SI, Figure S10B,C), but the atropine recoveries were also minimal (see Figure 3A). To improve the extraction recoveries with these solvents, just before extraction, the buckwheat cereals were first wetted with 2 mL of 0.05 M NaOH in water. Under alkaline conditions, atropine is predominantly not protonated and therefore, highly soluble in more nonpolar solvents, such as butyl acetate and ACN.^27^ Therefore, when butyl acetate or ACN is used as extraction solvent after prewetting the buckwheat cereals, recoveries of approximately 80% were obtained (see Figure 3A). In addition, matrix coextraction in the organic solvents was still minimized (see SI, Figure S10D,E). With the prewetting procedure and butyl acetate, already after 5 min, 80% recovery of atropine was reached (see SI, Figure S12), which is comparable with the reported recoveries obtained in much more intensive and longer extraction and cleanup procedures for tropane alkaloid analysis.^10,12,25^ As butyl acetate forms a two layer system with water, which is required for the PI-LPME in step II of the modular workflow, butyl acetate was selected as the extraction solvent for subsequent experiments.

#### Paper-Immobilized Liquid-Phase Microextraction: Extraction

After the SLE was optimized for sample extraction, a low-cost PI-LPME protocol to capture atropine from extracts was developed. Chromatography paper, fixed inside the 3D-printed PI-LPME frame (iv), was prewetted with 0.1% (v/v%) formic acid in water. Due to differences in capillary pressure, butyl acetate does not wick in the paper to replace the aqueous solution. As the paper in the PI-LPME frame (iv) was wetted with only 10 μL of 0.1% (v/v%) formic acid and the extraction was performed in 1.8 mL of butyl acetate, the phase ratio aqueous: organic layer was 1:180. After 18 min of PI-LPME, the atropine concentration in the butyl acetate was reduced from 53 to 29 ng mL^–1^, resulting in an estimated tropane alkaloid removal of 45%. (see Figure 3B). As the phase ratio is 1:180, this recovery results in an enrichment of about a factor of 80. The tropane alkaloids can be eluted from the paper with 0.1% (v/v%) formic acid in water (see SI, Figure S13), and the recovered amount of atropine showed good correspondence with the amount removed.

#### Paper-Immobilized Liquid-Phase Microextraction: Elution

After the PI-LPME extraction for capturing atropine, the elution of atropine from the paper was optimized. The performances of RB and 0.1% (v/v%) formic acid in water as elution solvents were assessed. After 15 min of elution time, comparable atropine recoveries were obtained with both RB (77% ±3%, *n* = 3) and 0.1% (v/v%) formic acid in water (82% ±4%, *n* = 3) as elution solvents. As the icLFIA performance is optimal when its run with RB, of which the composition was optimized, RB as the elution solvent was chosen. After 2 min, already 60% of the atropine captured on the paper was eluted, which increased to 80% after 10 min (see Figure 3C). In later experiments, 10 min of PI-LPME elution was performed, as this was fit-for-purpose and longer elution times would only add to the total analysis time of the workflow.

### Bioreagent Characterization and icLFIA Detection

#### Bioreagent Characterization

With an icMI, the sensitivity and specificity of the monoclonal antibody (mAb) and conjugate used in the icLFIA were determined for seven different tropane alkaloids (see SI, Figure S14 and Table S3). For atropine in 0.01 M PBS in water containing 0.1% BSA and 0.02% Tween 20, the IC_50_ value was 0.79 ng mL^–1^. The lowest IC_50_ value for nonatropine tropane alkaloids was obtained for homatropine of 16.75 ng mL^–1^. This results in a cross-reactivity of 4.7%. All other nonlegislated tropane alkaloids and scopolamine-obtained IC_50_ values greater than 100 ng mL^–1^, thus, resulting in cross-reactivities below 0.8%. Only one mAb with a lower IC_50_ for atropine (0.05 ng mL^–1^) has been reported.^6^ However, this mAb had a far lower specificity, with cross-reactivities to three nonlegislated tropane alkaloids ranging between 20.8% and 71.4%. The present mAb therefore enables the specific detection of atropine.

#### icLFIA Detection

As the icMI results showed that the mAb is both sensitive and specific for atropine, an atropine-specific icLFIA was developed. After optimization, the sensitivity of the icLFIA was tested with atropine standard solutions in RB both in a 96-well plate format and by placing the icLFIA in a 3D-printed cassette (vii). IC_50_ values of 0.33 and 0.56 ng mL^–1^ were obtained (see Figure S15), indicating that the icLFIA performed comparably in the well and in the 3D-printed cassette.^6,10^

### Characterization of the Sample Preparation of the Modular Workflow

The applicability of the sample preparation of the modular workflow for different tropane alkaloids in cereals was evaluated by performing the complete workflow with atropine, scopolamine, homatropine, and anisodine spiked samples and measuring the extracts of all intermediate steps with LC-MS/MS. The SLE recoveries for all tropane alkaloids ranged between 66% and 79% (see Table 1). It can be observed that, for atropine, the highest SLE recovery was obtained, followed by homatropine, scopolamine, and anisodine. These results are in line with computational calculations, in which the calculated log(*P*) values in a 1-octanol/water system based on the tropane alkaloid structure is greatest for atropine, followed by homatropine, scopolamine, and anisodine (see Table 1).^28^ During the SLE, in which the tropane alkaloids are extracted with an apolar solvent, it can be expected that the highest SLE-recovery is obtained for the most apolar analyte, i.e., atropine.

**Table 1:** Evaluation of the Sample Preparation Workflow of the Prototype Immunoassay (*n* = 5)

tropane alkaloid	Log(*P*)^28^	SLE recovery (%, ±SD)	PI-LPME extraction removal (%, ±SD)	PI-LPME CF immobilized aqueous solution	PI-LPME elution recovery (%, ±SD)	PI-LPME CF running buffer	repeatability (RSD%)
atropine	2.19	79 (±3)	34 (±2)	60 (±4)	83 (±13)	2.5 (±0.5)	22
scopolamine	1.40	72 (±3)	59 (±6)	106 (±11)	89 (±11)	4.7 (±0.4)	14
homatropine	1.91	73 (±7)	47 (±4)	84 (±7)	89 (±5)	3.8 (±0.4)	11
anisodine	0.59	65 (±5)	60 (±4)	108 (±7)	94 (±11)	5.0 (±0.9)	20

During the PI-LPME extraction, between 34% and 60% of the tropane alkaloids were removed from the SLE extract and transferred to the immobilized acidic solution in the paper (Table 1). As there was a phase ratio of 1:180, these result in a PI-LPME CF to the aqueous solution immobilized in the paper ranging between 60 and 108. Here the highest concentration effect was observed for anisodine, followed by scopolamine, homatropine, and atropine. During the PI-LPME, the molecules partition between the organic extract and acidic paper. Therefore, it can be expected that the highest concentration effect is obtained for the most hydrophilic tropane alkaloid, i.e., anisodine.^28^ The CF of 60 for atropine from the crude cereal extract is somewhat lower than that of spiked butyl acetate, i.e., 80, indicating some matrix effect. The subsequent recovery of the PI-LPME elution ranged between 83% and 93%, which results in a final CF of the SLE-extract to RB via PI-LPME, ranging from 2.5 to 5.0 (see Table 1). The overall repeatability of the workflow ranged between 10% and 22%.

For on-site detection, the greenness and point-of-need applicability of the method is important. Therefore, these characteristics have been assessed using AGREEprep (see SI, Figure S16).^29^ In the AGREEprep report, it can be observed that the sample preparation workflow performs well on the criteria: size economy of the sample (criteria 5), energy consumption (criteria 8), and postsample preparation configuration for analysis (criteria 9). The workflow can be further improved by a reduction of the amount of hazardous solvents used (criteria 2) and a reduction in the amount of steps of the workflow (criteria 7). Here it must be stated that current icLFIAs that follow an extract and dilute sample preparation approach are not capable of detecting atropine at regulatory levels in buckwheat cereals.^10^ In addition, although the current modular workflow still employs a shaker during the SLE and stirrer during the PI-LPME, such agitation can also be performed manually or battery-driven stirrers and shakers can be used. Furthermore, in comparison to current analysis methods that can detect atropine in buckwheat cereals at regulatory levels (e.g., enzyme-linked immunosorbent assay or LC-MS/MS), these equipment requirements are minor.

### Validation of the Prototype Immunoassay

**Figure 4:**
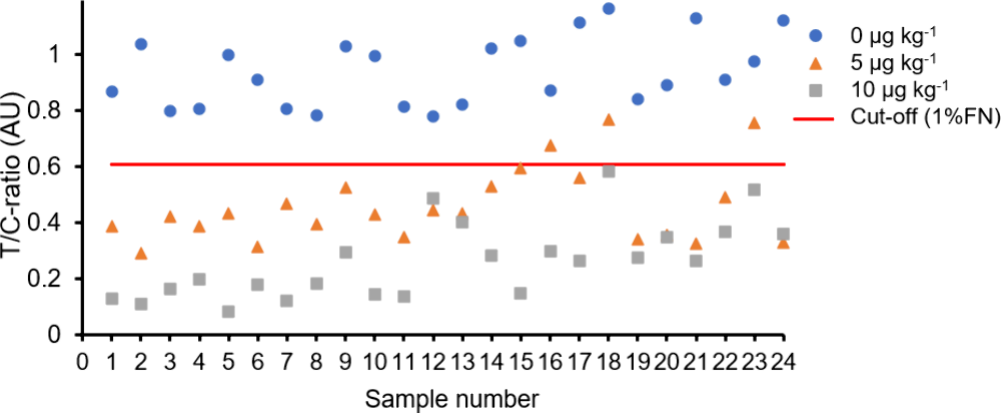
T/C ratios of the icLFIA after analyzing 24 blank and spiked buckwheat samples at 5 μg kg^–1^ (0.5 × maximum level) and 10 μg kg^–1^ (1.0 × maximum level) atropine with the modular workflow: T/C ratio for the blank samples (blue circles), 5 μg kg^–1^ samples (orange triangles), or 10 μg kg^–1^ samples (gray squares), and cutoff with an estimated false negative rate (FN) of 1% (red line).

The applicability of the modular workflow for tropane alkaloid detection at relevant levels with an icLFIA was demonstrated by validating it in-house with an atropine-specific icLFIA. The T/C ratios of the icLFIA after analyzing the buckwheat samples are shown in Figure 4. The cutoff value at an estimated 1% false negative rate is also shown. The data in Figure 4 show that the blank samples can be clearly discriminated from the samples contaminated at 10 μg kg^–1^ atropine, as all blank samples had a T/C ratio above the cutoff value with 1% false negative rate. Therefore, the estimated probability of false positive results was very low, 0.68%, even though the cutoff value was set at a 1% false negative rate, rather than the less stringent 5% recommended by EU legislation 2023/2783 (which would yield an estimated false positive rate of 0.09%). It should be noted that the estimated false negative and false positive rate are both depended on the established cutoff value. If for example the cutoff value is changed to reduce the estimated false negative rate, this will result in an increase of the estimated false positive rate, and vice versa. If a cutoff value with a 5% false negative rate is established based on the icLFIA results after analyzing the buckwheat cereals contaminated at half-maximum level (5 μg kg^–1^ atropine), the probability of a false positive result is still low, 2.03%.

Our reported sample-to-solution approach in combination with the icLFIA clearly allows for atropine detection in cereal-based products at regulatory levels, whereas, previously reported biosensors for tropane alkaloid detection in cereal-based products, as well as our icLFIA without sample pretreatment, were not sensitive enough (LODs: >10 μg kg^–1^).^10^ A lab-based indirect competitive enzyme-linked immunosorbent assay method has been demonstrated for the screening of atropine in milled buckwheat cereals at regulatory levels (LOD: 0.85 μg kg^–1^), however, this method is not easily applied at the point-of-need.^6^ The current LC-MS/MS methods expectedly have superior detection limits (LODs 0.05–2 μg kg^–1^) compared to all screening methods, but again, LC-MS/MS methods are lab-based and much more expensive.^4^ The modular workflow eventually enables a monitoring strategy with fast point-of-need screening for atropine, in which only the samples screened as positive (suspect) are send to a laboratory for confirmatory analysis, e.g. by LC-MS/MS. As the sample preparation of the modular workflow has been demonstrated for scopolamine, and several antibodies with similar sensitivities for scopolamine as our antibody for atropine have been reported, it should be feasible to integrate these antibodies on an icLFIA to expand the modular workflow toward scopolamine detection.^6,30^

## Conclusion

An on-site applicable modular workflow was developed that allows for reliable point-of-need detection of atropine in buckwheat cereals at EU-regulatory levels. The novel SLE-protocol, based on the pH-dependency of tropane alkaloids, minimizes matrix coextraction, while a newly developed paper-immobilized liquid phase microextraction (PI-LPME) concentrates the tropane alkaloids. This sophisticated sample extraction and cleanup procedure was demonstrated for atropine, scopolamine, anisodine, and homatropine. It is rapid, cheap, and easy, and both eliminates matrix effects to a great extent and enhances the sensitivity and selectivity of the method. The 3D-printed SLE-filter attachment, PI-LPME frame, and PI-LPME holder integrated with an icLFIA in a 3D-printed cassette enhance reproducibility, user-friendliness, and the safety of the end-users. Furthermore, execution of the complete modular workflow requires only 50–80 min. The modular workflow was validated by analyzing 24 blank and spiked buckwheat cereal samples with 5 and 10 μg kg^–1^ atropine. A cutoff value was established with an estimated false negative rate of 1% and estimated false positive rate of 0.68%. As the complete sample preparation of the modular workflow is also demonstrated for scopolamine, and specific scopolamine antibodies exist, it should be feasible to also detect scopolamine in buckwheat cereals at regulatory levels by including a corresponding icLFIA in the modular workflow. Furthermore, our modular workflow can be further improved by expanding its application toward different food matrices that require even lower detection levels, such as cereal-based infant food. In order to reach such detection levels, the modular workflow could be further optimized, for example, by increasing the dimensions of the PI-LPME (e.g., increasing PI-LPME paper size). In addition, integrating the modular workflow with different detection approaches instead of an icLFIA, could be considered. The current modular workflow is expected to become a useful tool for local authorities, small companies, and laboratories as a prescreening method for atropine detection of buckwheat cereals at the point-of-need. Furthermore, in low-income countries where regular LC-MS/MS analysis might be unavailable, the workflow can enhance food safety even more. In addition, the modular workflow has the potential to be expanded to the on-site detection of many other analytes with a pH-dependent partition coefficient in the fields of, for example, forensics, biomedicine, and food safety.

## Supporting Information Available

Chemical structure and information on seven tropane alkaloids; Additional experimental details, materials, and methods, including construction of 3D-printed devices; Extraction recovery after SLE and PI-LPME; Sensitivity and specificity of the atropine specific immunoassay using an icLFIA or icMI; Signal intensity of the T-line and C-line of the icLFIA after measuring diluted solid–liquid extracts; AGREEprep assessment of the greenness of the sample preparation (PDF)
