# Supplementary material for: Modular Point-of-Need Tropane Alkaloid Detection at Regulatory Levels: Combining Solid–Liquid Extraction from Buckwheat with a Paper-Immobilized Liquid-Phase Microextraction and Immuno-Detection in Interconnectable 3D-Printed Devices
Source: Anal Chem. 2024 Oct 4;96(41):16462–8. doi: 10.1021/acs.analchem.4c04811 (PMC11483449; doi:10.1021/acs.analchem.4c04811)
Supplement: Supplementary file 1 — ac4c04811_si_001.pdf [file ac4c04811_si_001.pdf]

`

Supporting Information

Modular point-of-need tropane alkaloid detection at regulatory levels: combining solid-liquid extraction from buckwheat with a paper immobilized liquid phase microextraction and immuno-detection in interconnectable 3D-printed devices

Ids B. Lemmink [1, 2]; Linda Willemsen [2]; Erik Beij [2]; Toine F.H. Bovee [2]; Han Zuilhof [1, 3]; Gert IJ. Salentijn [1, 2*]

[1] *Laboratory of Organic Chemistry, Wageningen University & Research, Stippeneng 4, 6708 WE, Wageningen, The Netherlands*

[2] *Wageningen Food Safety Research, Wageningen University & Research, Akkermaalsbos 2, 6708 WB Wageningen, The Netherlands*

[3] *School of Pharmaceutical Sciences and Technology, Tianjin University, 92 Weijin Road, Tianjin, 300072, China.*

*corresponding author; gert.salentijn@wur.nl

`

Table of Contents

**1. Experimental section**		
Protocol S1	Chemicals and consumables	Page S3
Protocol S2	Instrumental setups	Page S4
Protocol S3	Experimental procedures	Page S5-8

**2. Figures and tables**		
Figure S1	Chemical structure and information of seven tropane alkaloids	Page S9
Figure S2	Step-by-step procedure of the modular workflow for tropane alkaloid detection in buckwheat cereals at the point-of-need.	Page S10
Figure S3	Overview of the development, optimization, and characterization of the modular workflow for tropane alkaloid detection in buckwheat cereals	Page S11
Table S1	HPLC-MS/MS acquisition parameters for atropine, scopolamine, homatropine and anisodine	Page S12
Figure S4	HPLC-MS/MS calibration curve for atropine, scopolamine, homatropine and anisodine	Page S13
Table S2	Prusa Original i3 MK3S+ slicer settings used during printing of the solid liquid extraction-filter attachment and indirect competitive lateral flow immunoassay cassette	Page S14
Figure S5	Schematic overview of the indirect competitive lateral flow immunoassay design	Page S15
Figure S6	Construction and operation of the solid liquid extraction-filter attachment	Page S16
Figure S7	Construction and operation of the paper-immobilized liquid phase microextraction frame	Page S17
Figure S8	Construction and operation of the paper-immobilized liquid phase microextraction holder	Page S18
Figure S9	Construction and operation of the indirect competitive lateral flow immunoassay cassette	Page S19
Figure S10	Extract composition after a solid liquid extraction of buckwheat cereals with different extraction solvents	Page S20
Figure S11	Signal intensity of the T-Line and C-line of the indirect competitive lateral flow immunoassay after diluting the extract	Page S21
Figure S12	Atropine recovery with different extraction times during the solid liquid extraction	Page S22
Figure S13	Amount of atropine removed from the spiked butyl acetate and recovered from the paper after the paper immobilized liquid phase microextraction with varying extraction times	Page S23
Figure S14	Dose-response curves of the atropine-specific antibody and atropine-bovine serum albumin conjugate in an indirect competitive microsphere-based immunoassay for seven tropane alkaloids	Page S24
Table S3	Sensitivity and specificity of the atropine-specific antibody and atropine-bovine serum albumin conjugate in the indirect competitive microsphere-based immunoassay for seven tropane alkaloids	Page S25
Figure S15	Dose-response curves of the atropine-specific antibody and atropine-bovine serum albumin conjugate in an indirect competitive lateral flow immunoassay run in 96-well plate or 3D-cassette for atropine	Page S26
Figure S16	Assessment of the greenness of the sample preparation of the modular workflow using AGREEprep	Page S27
References		Page S28

1. Experimental section

Protocol S1 Chemicals and consumables

S1.1 Chemicals

Atropine (>99%), tropine (97%), scopolamine hydrobromide (>98%), homatropine hydrobromide (>99%), and butyl acetate (>99%) were purchased from TCI Europe (Zwijndrecht, Belgium), anisodamine (>98%), and anisodine hydrobromide (>99%) from Phytolab (Vestenbergsgreuth, Germany), and formic acid (FA, HPLC-grade), Tween 20, and bovine serum albumin (BSA, ≥98%) from Sigma Aldrich Co. (Saint Louis, USA). Acetonitrile (ACN, LC-MS grade), and methanol (MeOH, LC-MS grade) were obtained from Actu-all Chemicals (Oss, The Netherlands), sodium hydroxide (NaOH, pellets), and PBS tablets from Merck KGaA (Darmstadt, Germany), and carboxylic paramagnetic MagPlex microspheres No. 55 from Luminex Corp. (Austin, USA). The goat anti-mouse phycoerythrin conjugated immunoglobulin G was purchased from Moss (Pasadena, USA), and 'Spezial Schwartz 4' carbon nanoparticles from Degussa AG (Frankfurt, Germany). The goat anti-mouse IgG in PBS (pH 7.6) (1.2 mg mL^{-1}; AffiniPure F(ab')2 Fragment GAM IgG Fcγ) was purchased from Jackson Immunoresearch Laboratories Inc. (West Grove, USA), while the monoclonal anti-atropine antibody (anti-atropine mAb), and atropine-BSA conjugate were obtained from Jiangnan University (Wuxi, China). The running buffer (RB) for the icLFIA as applied in this work is 0.01 M PBS in water containing 1% BSA and 0.05% Tween 20. The PBST is 0.01 M PBS in water containing 0.1% BSA and 0.02% Tween 20. Deionized water was from a Milli-Q direct ultrapure water system (Millipore, USA).

S1.2 Consumables

Polystyrene 96-well microplates with flat bottom and Whatman cellulose Chromatography paper Chr 1 were purchased from Sigma Aldrich Co. (Saint Louis, USA), protein LoBind safe-lock tubes 1.5 mL, and 2.0 mL (PCR clean) from Eppendorf Corporate (Hamburg, Germany), 5 mm stainless steel grinding balls from Avantor (Radnor Townsship, USA), and tea filter bags (pore size ±50 µm) from Simon Levelt (Utrecht, The Netherlands). The 16 mL glass screw vails were purchased from Thermo Fisher Scientific (Waltham, USA), the 2.5 mL syringes from TERUMO Europe N.V. (Leuven, Belgium), and the UniSart 95 CN nitrocellulose membranes from Sartorius (Gottinghem, Germany). Plastic backing cards (30 cm × 6 cm), glass fiber (25.4 cm × 30.48 cm), and sample pads (21 cm × 29.7 cm) were purchased from Kenosha (Amstelveen, The Netherlands).

S1.3 Reference material preparation

As tropane alkaloids enter the food chain through cross-crop contamination of weeds during the harvest of cereals (i.e., a small amount of plant material containing very high amount of toxins, contaminating a large volume of toxin-free crops), and the tropane alkaloids are subsequently distributed in the food product through milling, real samples are the result of a blending process. Since, certified reference materials for tropane alkaloids are not commercially available, all materials with atropine, scopolamine, homatropine, and anisodine were prepared in-house by spiking milled buckwheat cereal samples. All the prepared tropane alkaloid solutions were analyzed with HPLC-MS/MS for quantification of atropine, scopolamine, homatropine, and anisodine concentrations according to external standard calibration curves (see SI, Figure S4). Twenty-four different milled (certified-organic) buckwheat cereals were bought from local suppliers. When two buckwheat cereal samples came from the same supplier it was ensured they had a different batch number. All buckwheat cereals were analyzed with a in-house validated LC-MS/MS method, to ensure they were not contaminated with any tropane alkaloids (i.e., that they were blanks).[1] One gram of buckwheat cereals was precisely weighed and spiked with different concentrations of atropine, scopolamine, homatropine, or anisodine in 100µL of ACN (50-500 ng mL^{-1}). The buckwheat cereals were dried for at least 24h and thoroughly mixed before further use.

`

Protocol S2 Instrumental setups

S2.1 HPLC-MS/MS

The HPLC-MS/MS method was adapted from the method described by Jakabová *et al.*[2] A high performance liquid chromatograph (1220 Infinity II LC; Agilent Technologies; Santa-Clara, USA), equipped with a ZORBAX SB-C18 column (3.0 × 250 mm, 5 µm; Agilent Technologies; Santa-Clara, USA) and a SPD-M20A photodiode array detector (Shimadzu Corporation; Kyoto, Japan) was coupled to an ion trap mass spectrometer (Finnigan LXQ; Thermo Fisher Scientific; Waltham, USA). The column oven was set at 50 °C. The mobile phase consisted of 0.1% (v/v%) formic acid in both water (mobile phase A) and MeOH (mobile phase B), and the flow rate was 0.6 mL min^{-1}, using a gradient of: 0-1 min 10% B; 1-11 min linear ramping to 90% B; 11-16 min 90% B; 16-16.10 min linear decrease to 10% B; 16.10-21 min re-equilibration at 10% B. Mass spectrometry was performed in positive multiple reaction monitoring (MRM) mode (see SI, Table S1, for settings) with an atomizer flow rate of 3 $L{\cdot}min^{-1}$, a heating gas flow rate of 10 $L{\cdot} min^{-1}$, a drying gas flow rate of 10 $L{\cdot}min^{-1}$, DL temperature 275 °C, ion source interface voltage of 3.5 kV, and heating block temperature of 400 °C.

S2.2 Microsphere immunoassay

A MAGPIX planar array analyzer (Luminex; Austin, USA) equipped with xPONENT 4.3 software (Luminex; Austin, USA) was used for a read-out of the fluorescence of the indirect competitive microsphere immunoassay (icMI). The acquisition volume was set at 50 µL. GraphPad Prism version 10 (Domatics; Boston, USA) was used for data processing and five-parameter logistic curve fitting.

S2.3 Development, 3D-design, and 3D-printing of interconnectable sample preparation and detection tools

The modular workflow consisted of a combination of readily-available laboratory consumables and 3D-printed devices. Computer-aided design software SOLIDWORKS Education Edition 2021-2022 (SOLIDWORKS Corp; Waltham, USA) was used for designing the 3D-printable parts and converting them to printable STL. files. The PI-LPME frame and holder were printed with a SLA-printer Form3 (FormLabs; Summerville, USA) at 100 µm layer resolution using a proprietary clear resin (Type V4, FormLabs; Summerville, USA). A FDM Original Prusa i3 MK3S+ printer (Prusa Research; Praque, Czech Republic) was used to print the SLE-filter attachment and icLFIA cassette using biobased PLA-filament (PolyLite PLA Pro; Polymaker; Utrecht, NL, Table S2, for settings).

`

Protocol S3 Experimental procedures

S3.1 Development of the sample preparation

S3.1.1 Solid-liquid extraction

10 mL ACN, 0.1% (v/v%) formic acid in water, and butyl acetate were initially tested as extraction solvents. Eventually, extraction solvent combinations of 2 mL 0.05 M NaOH in water with either 10 mL butyl acetate or 10 mL ACN were tested. The NaOH solution was added first and after 20 seconds of manual shaking ACN or butyl acetate was added. Subsequently, the extracts were shaken with an overhead shaker (Heidolph Reax 2; Heidolph Instruments GmbH & Co. KG; Schwabach, Germany) for 30 min at room temperature. After 30 min of extraction, the extract was left to settle by gravity for 5 min. The tropane alkaloid concentration in the extract was determined with LC-MS/MS. The extraction recovery was calculated following:

(1) $$\text{Extraction recovery (\%)} = \frac{[\text{TA}]_{\text{extract}} * V_{\text{extract}}}{[\text{TA}]_{\text{spike}} * V_{\text{spike}}} * 100\%$$

Where $[TA]_{extract}$ = measured tropane alkaloid concentration (ng mL^{-1}) in the SLE extract, $V_{extract}$ = volume (mL) of the SLE extract, $[TA]_{spiked}$ = tropane alkaloid concentration (ng mL^{-1}) in the spike solution, and V_{spiked} = volume (mL) of the spike solution.

After the optimal extraction solvent conditions were determined, the extraction time was optimized by repeating the extraction procedure described above with the solvent combination 2 mL 0.05 M NaOH in water with 10 mL butyl acetate for 5, 15, and 30 min.

S3.1.2 Paper-immobilized liquid phase microextraction: extraction

For the PI-LPME extraction, the extraction time was optimized. A 2 mL Eppendorf tube with a micro stirring bar was filled with 1.62 mL butyl acetate and spiked with 180 µL of 500 ng mL^{-1} atropine. Paper (23 mm × 5 mm) was pre-wetted with 10 µL 0.1% (v/v%) formic acid in water. Subsequently, the paper was inserted in the 2 mL Eppendorf tube and stirred at 500 rpm using a magnetic stirring plate (LABOTECH EM 3300T; Zwolle, The Netherlands). After 2, 4, 6, 8, 10, 12, 14, 16, or 18 min, the paper was removed from the 2 mL Eppendorf tube and subsequently, extracted overnight by inserting it in an 1.5 mL Eppendorf tube containing 200 µL of 0.1% (v/v%) formic acid in water. To the spiked butyl acetate before and after the PI-LPME, 0.1% (v/v%) formic acid in water (phase ratio 1:5) was added, and the aqueous phase was measured by LC-MS/MS. Additionally, the PI-LPME paper was eluted with 200 µL 0.1% (v/v%) formic acid in water overnight, and this was measured with LC-MS/MS. The percentage of tropane alkaloids removed from the organic solution during the PI-LPME was calculated following:

(2) $$\text{Removal (\%)} = \frac{[\text{TA}]_{\text{before PI-LPME}} - [\text{TA}]_{\text{after PI-LPME}}}{[\text{TA}]_{\text{before PI-LPME}}} * 100\%$$

Where $[TA]_{before\ PI\text{-}LPME}$ = tropane alkaloid concentration (ng mL^{-1}) recovered with acidic water from the spiked butyl acetate before the PI-LPME, and $[TA]_{after\ PI\text{-}LPME}$ = tropane alkaloid concentration (ng mL^{-1}) recovered with acidic water from the spiked butyl acetate after the PI-LPME. The concentration factor (CF) of the PI-LPME to the aqueous solution immobilized in the paper was calculated following:

(3) $$\text{CF}_{\text{immoblized aqeous solution}} = \frac{V_{\text{org}}}{V_{\text{aq}}} * \frac{\text{Removal (\%)}}{100\%}$$

Where V_{org} = volume (mL) of organic solution during PI-LPME, V_{aq} = volume (mL) of aqueous solution immobilized in the paper, and Removal (%) = tropane alkaloids removed (%) from the organic solution during the PI-LPME as calculated in Equation 2.

`

S3.1.3 Paper immobilized liquid phase microextraction: elution

For the PI-LPME elution, elution time, and elution solvent were optimized. The optimal elution solvent was determined by spiking dry paper (23 mm × 5 mm) with 10 µL of 1000 ng mL^{-1} atropine in 0.1% (v/v%) formic acid in water. The paper was left to dry for 20 min. The paper was then submerged in 200 µL of 0.1% (v/v%) formic acid in water or RB and extracted for 15 min. The concentration of atropine in the extract was determined by LC-MS/MS. The elution recovery was calculated following:

(4) $$\text{Elution recovery (\%)} = \frac{[TA]_{elution} * V_{elution}}{[TA]_{spike} * V_{spike}} * 100\%$$

Where $[TA]_{elution}$ = tropane alkaloid concentration (ng mL^{-1}) in the PI-LPME elution solvent, $V_{elution}$ = volume (mL) of the PI-LPME elution solvent, $[TA]_{spike}$ = tropane alkaloid concentration (ng mL^{-1}) in the spike solution, and V_{spike} is the volume of the spike solution (mL).

To determine the minimal elution time, dry paper was spiked and dried, following the procedure as described above. After drying, the paper was extracted with 200 µL of RB for 2, 4, 6, 8, and 10 min, and overnight. The concentration of atropine in the extract was determined by LC-MS/MS and the extraction recovery calculated following Equation 4.

S3.2 Immunoassay characterization and detection

S3.2.1 Bioreagent characterization

The anti-atropine mAb and atropine-BSA conjugate were first evaluated for their sensitivity and specificity for seven tropane alkaloids in an icMI. The protocol for the icMI measurements has been adapted from the protocol described by Zou *et al.*[3] The icMI was performed in a polystyrene 96-well microplate with flat bottom. The atropine-BSA conjugates were immobilized on paramagnetic MagPlex microspheres (No. 55) with a EDC/Sulfo-NHS coupling as described by Angeloni *et al.*[4] Ten-fold serial dilutions of standards from atropine, scopolamine, homatropine, aposcopolamine, anisidine, anisodamine, and tropine (ranging from 0.01 ng mL^{-1} to 1000 ng mL^{-1}) were added to individual wells (100 µL per well). Subsequently, the icMI was performed by adding 10 µL of anti-atropine monoclonal antibody (4.5 µg mL^{-1}). The plate was incubated at room temperature for 20 min with moderate shaking (400 rpm), and then washed with PBST buffer (0.01M PBS, 0.1% BSA, 0.02% tween 20; 100 µL/well) twice using a magnetic plate separator (Sigma-Aldrich Co.; St. Louis, USA). The fluorescently labeled secondary antibody, R-phycoerythrin coupled goat-anti-mouse IgG, was then added to the plate (100 µL per well) to a final concentration of 2 mg L^{-1}. After another 20 min incubation, followed by two washing steps with PBST, the microspheres were resuspended in 100 µL of PBST. The median fluorescence intensity (MFI) of 100 events was measured with the MAGPIX™ system. The specificity was expressed in terms of cross-reactivity (CR):

(5) $$CR = \frac{IC_{50}[\text{atropine}]}{IC_{50}[\text{competitor}]} \times 100\%$$

Where CR = cross reactivity (%), IC_{50}[atropine] = half-maximum inhibitory concentration of atropine (ng mL^{-1}), and IC_{50}[competitor] = half-maximum inhibitory concentration of another tropane alkaloid (ng mL^{-1}).[5]

S3.2.2 Indirect competitive lateral flow immunoassay detection

After the sensitivity and specificity of the anti-atropine mAb and atropine-BSA conjugate was determined, an icLFIA was developed, optimizing the type of NC-membrane, RB-composition, drying buffer composition, and primary antibody, secondary antibody, atropine-BSA conjugate, and donkey anti-goat antibody concentrations (see SI, Figure S5). ATR-BSA conjugate (0.25 mg mL^{-1}) for use on the test line (T-line) and donkey anti-goat IgG antibody (0.15 mg mL^{-1}) for use on the control line (C-line) were dissolved in 0.01 M PBS buffer, and subsequently sprayed onto nitrocellulose (NC) membranes (1 µL per strip). Donkey anti-mouse IgG antibody was coupled to carbon nanoparticles as described by Ross *et al.*[6] Hyoscyamine-specific antibody (22.5 µg mL^{-1}) for the antibody pad and donkey anti-goat IgG antibody

`

coupled to carbon nanoparticles for the labeled secondary-antibody pad were dissolved in 0.01 M PBS buffer with 1% BSA and 0.01% Tween 20, and subsequently sprayed onto glass fiber (12 µL per strip). The antibody pad, conjugate pad and NC membrane were glued on plastic backing cards. Wicking pads were laminated onto the top of the NC membrane to ensure constant flow (see Figure S5). Atropine calibration standards of 0, 0.1, 0.5, 1, 5, and 10 ng mL^{-1} in RB were prepared to test the sensitivity of the developed icLFIAs in both a 96-well plate and in a 3D-printed icLFIA cassette. The icLFIAs were wetted with 100 µL of calibration standard solution and allowed to develop for 15 min. After 15 min, the intensities of the T- and C-line were quantified with a colorimetric reader (Cubereader; Chembio Diagnostics GmbH; Berlin, Germany). The cube-reader software was used to obtain grey scale intensity values for each peak, across the length of the icLFIA. The grey scale intensity results were normalized by dividing the T-line and C-line intensity, to obtain the T/C-ratio.[7]

S3.3 Characterization of the sample preparation of the modular workflow

The performance of the complete sample preparation of the modular workflow with matrix was characterized for atropine, scopolamine, homatropine and anisodine, by preforming the complete workflow with spiked buckwheat cereals and measuring the tropane alkaloid concentration at each intermediate step. Spiked buckwheat cereals were extracted for 30 min with NaOH (0.05M) in water and butyl acetate, as described in section S3.1.1. Three mL of extract was collected. One mL of the extract was evaporated with a gentle nitrogen flow and redissolved in 1 mL of 0.1% (v/v%) formic acid in water to be analyzed with LC-MS/MS. 1.8 mL of the remaining extract was transferred towards a 2 mL Eppendorf tube for a 20 min PI-LPME as described in section S3.1.2. After 20 min, the PI-LPME paper was removed and 1 mL of the residual extract was evaporated with a gentle nitrogen flow and redissolved in one mL 0.1% (v/v%) formic acid in water to be analyzed with LC-MS/MS. After the PI-LPME, the PI-LPME paper was extracted for 15 min with 200 µL of RB as described in section S3.1.3. Following, the RB was analyzed by LC-MS/MS. For each tropane alkaloid, the repeatability of the entire sample preparation workflow was calculated as the relative standard deviation (RSD%, n = 5) of the final tropane alkaloid concentration in RB. The final CF of the SLE-extract to RB, via PI-LPME, was calculated following:

$$\text{(6)} \qquad CF_{RB} = \frac{[\text{tropane alkaloid}]_{RB}}{[\text{tropane alkaloid}]_{SLE}}$$

Where $[\text{tropane alkaloid}]_{RB}$ = tropane alkaloid concentration in RB and $[\text{tropane alkaloid}]_{SLE}$ = tropane alkaloid concentration in SLE-extract.

S3.4 Validation of the modular workflow with an atropine-specific icLFIA

The modular workflow with an atropine-specific icLFIA was validated for the detection of atropine in milled buckwheat products by measuring twenty-four blank and twenty-four spiked buckwheat samples at both 0.5× maximum level (5 µg kg^{-1}) and maximum level (10 µg kg^{-1}), distributed over three consecutive days. Each sample was analyzed following Protocol S1 (see SI). After analyzing the samples with the immunoassay, the obtained T/C ratios were used to establish a cut-off value at which the false negative rate was 1% ($\beta = 0.01$) or 5% or ($\beta = 0.05$). The cut-off value was calculated following:

$$\text{(7)} \qquad \text{Cut-off value} = \bar{x}_{5/10} + \text{t-value}_{0.01/0.05} \times SD_{5/10}$$

Where $\bar{x}_{10}$ = average T/C ratio after analysis of the buckwheat samples spiked at 5 or 10 µg kg^{-1} atropine, t-$\text{value}_{0.01/0.05}$ = t-value for a one tailed t-test with false negative rate of 1% or 5% with 23 degrees of freedom, and $SD_{5/10}$ = standard deviation of T/C-ratios after analysis of the buckwheat samples spiked at 5 or 10 µg kg^{-1} atropine.

With the calculated cut-off value, the t-value corresponding to the event that a T/C ratio of a blank buckwheat sample is below the cut-off value and thus erroneously classified as positive was calculated. The t-value on a false positive result was calculated following:

`

(8) $$\text{t-value false positive} = \frac{\bar{x}_{\text{blank}} - \text{cut-off value}}{\text{SD}_{\text{blank}}}$$

Where $\bar{x}_{blank}$ = average T/C ratio after the analysis of blank buckwheat samples, cut-off value = cut-off value as calculated in equation 7, and SD_{blank} = standard deviation in T/C-ratios after the analysis of blank buckwheat samples.

With the t-value, the probability on a false positive result was calculated for a one-tailed t-distribution with 23 degrees of freedom.

2. Figures and tables

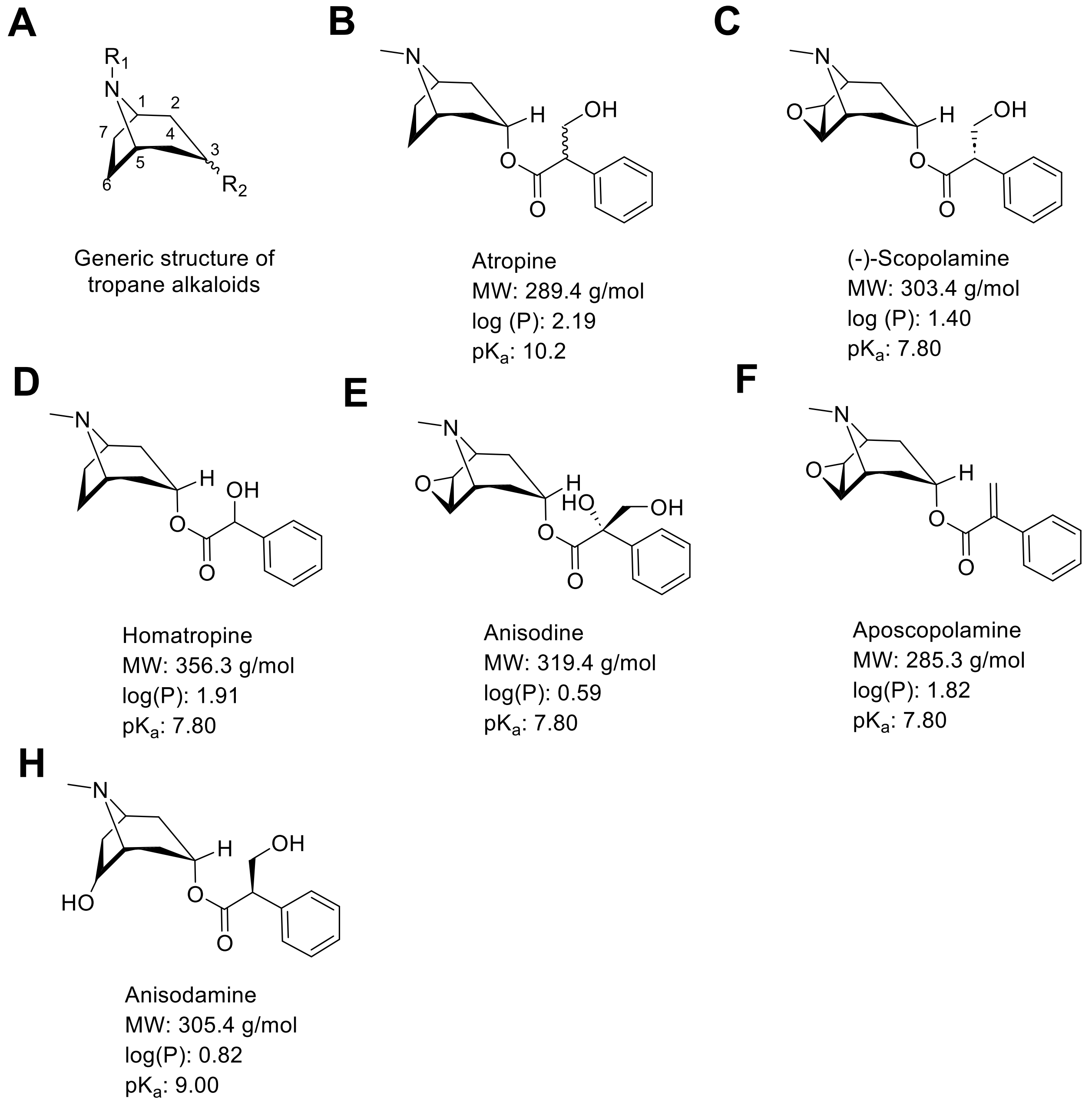

Figure S1. Chemical structure and chemical information of seven tropane alkaloids: molecular weight (MW), predicted Log(P) in 1-octanol/water system, and predicted pK_a-values.[8–10] All tropane alkaloids are characterized by a two-ringed structure with a piperidine and pyrrolidine ring sharing two carbon atoms and one nitrogen atom. Atropine is a racemic mixture of (+)-hyoscyamine and (-)-hyoscyamine. **(A)** Generic structure of tropane alkaloids, **(B)** Atropine, **(C)** Scopolamine, **(D)** Homatropine, **(E)** Anisodine, **(F)** Aposcopolamine, and **(H)** Anisodamine.

`

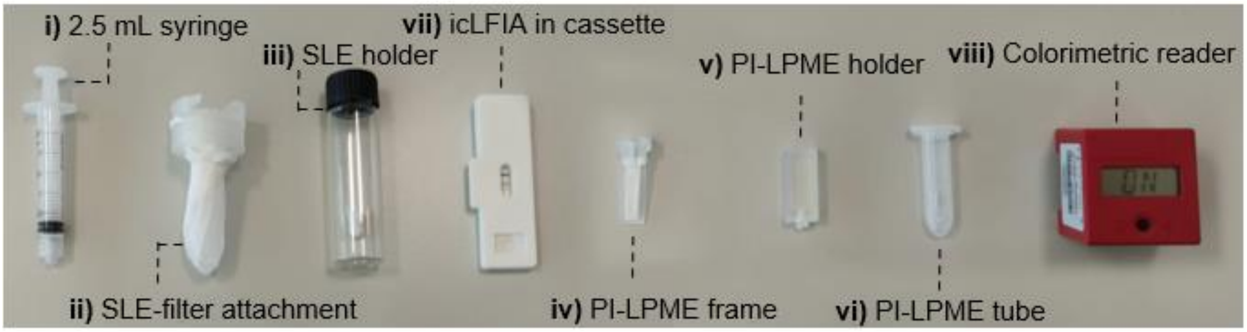

Overview of all components of the developed immunoassay, and their corresponding identification number (as used throughout the protocol): i) 2.5 mL syringe, ii) SLE-filter attachment, iii) SLE holder, iv) PI-LPME frame, v) PI-LPME holder, vi) PI-LPME tube, vii) icLFIA in cassette and viii) Colorimetric reader

Workflow of the biosensor for tropane alkaloid detection

<u>Workflow step i) Solid liquid extraction (SLE) – Duration: 30 min</u>

1. Place one gram of buckwheat cereals in the SLE holder (iii)
2. Add 2 mL of 0.05M NaOH in water
3. Shake vigorously for 10 seconds
4. Add 10 mL of butyl acetate
5. Extract the buckwheat cereals in the SLE holder (iii) for 30 minutes by overhead shaking
6. Click the 2.5 mL syringe (i) in the SLE-filter attachment (ii) and collect 1.8 mL of SLE extract
7. Fill the PI-LPME tube (vi) with 1.8 mL of the SLE extract

<u>Workflow step ii) Paper-immobilized liquid-phase microextraction (PI-LPME) – Duration: 20 min</u>

8. Pre-wet the paper in the PILPME frame (iv) with 10 µL of 0.1% (v/v%) formic acid in water
9. Insert the PILPME frame (iv) in the PILPME tube (vi) which contains a micro stirrer
10. Stir for 20 minutes

<u>Workflow step iii) PI-LPME elution – Duration: 10 min</u>

11. Fill the PI-LPME holder (v) with 200 µL of running buffer (0.01 M PBS in water containing 1% BSA and 0.05% Tween 20)
12. Insert the PI-LPME frame (iv) in the PI-LPME holder (v) and wait for 10 minutes
13. Unplug the PI-LPME holder (v) and connect it to the icLFIA cassette (vii)
14. Move the PI-LPME frame (iv), several times up and down in the PI-LPME holder (v) to press out the running buffer

<u>Workflow step iv) indirect competitive lateral flow immunoassay (icLFIA) detection</u>

15. Let the icLFIA run for 15 minutes
16. Attached the colorimetric reader (viii) to the icLFIA cassette (vii)
17. Measure the intensity of the test and control line by pressing the black button of the colorimetric reader (viii) three consecutive times

Figure S2. Step-by-step protocol of the modular workflow for tropane alkaloid detection in buckwheat cereals at the point-of-need.

`

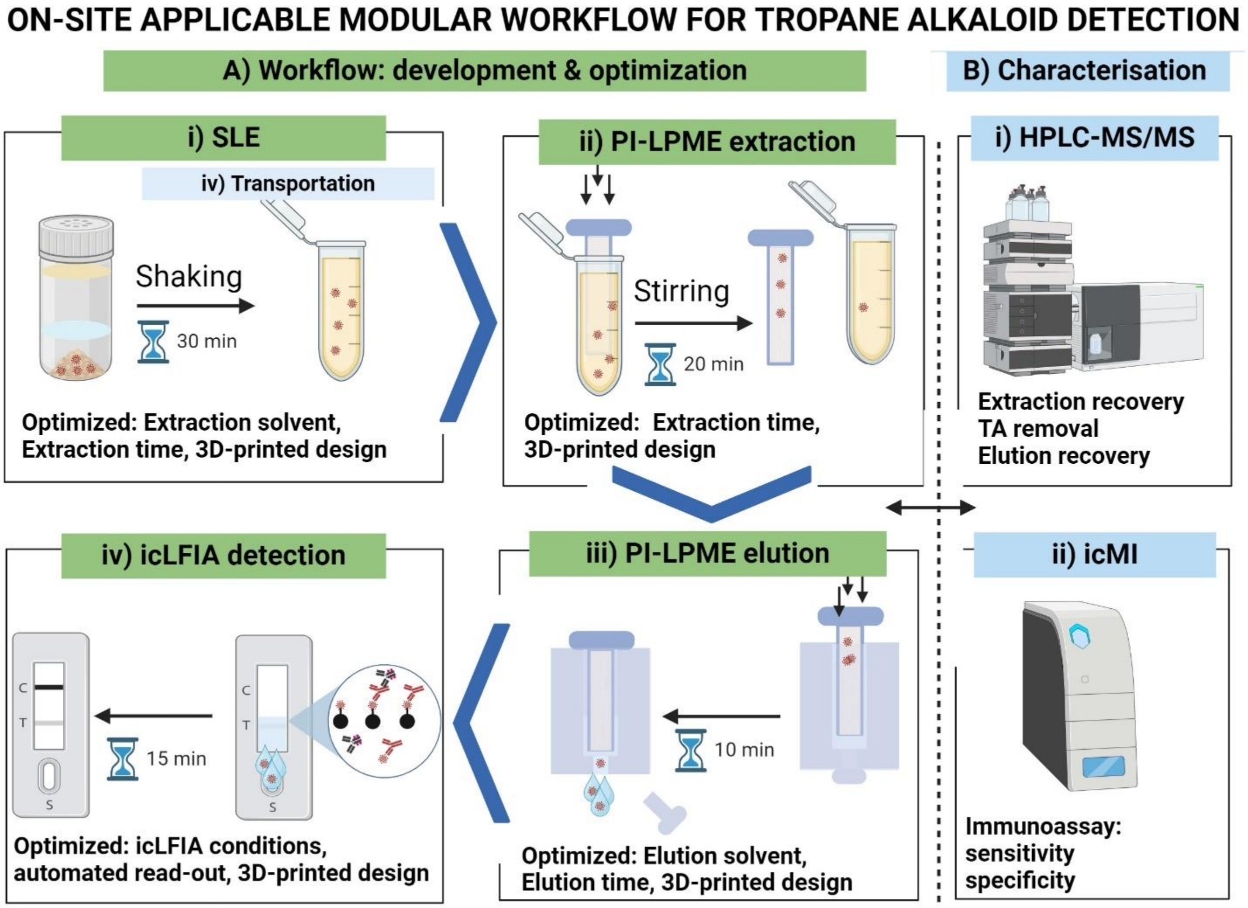

Figure S3. Overview of the development and optimization of the modular workflow for tropane alkaloid-detection in buckwheat cereals (created with biorender.com). (A) The four steps of the modular workflow that were developed and optimized: Step I) SLE, for which the extraction solvent composition and extraction time were optimized; Step II) PI-LPME extraction, for which the extraction time was optimized; Step III) PI-LPME elution, for which the elution solvent composition and elution time were optimized; Step IV) icLFIA: for which the icLFIA conditions and automated read-out were optimized. In all steps of the workflow, 3D-printed devices were made to enhance user-friendliness. (B) Techniques applied during development and optimization of the modular workflow: I) high pressure liquid chromatography-tandem mass spectrometry (LC-MS/MS) used for the characterization of workflow step I-III II) indirect competitive microsphere-based immunoassay (icMI) used for the characterization of workflow step iv.

`

Table S1. LC-MS/MS acquisition parameters for the four tropane alkaloids.

Compound	Precursor ion (m/z)	Product ion (m/z)	Collision energy (eV)	Retention time (min)
Atropine	290.2	124.0	25	7.47
Scopolamine	304.2	138.0	20	6.40
Homatropine	276.2	93.0	25	6.41
Anisodine	320.2	138.0	20	5.08

`

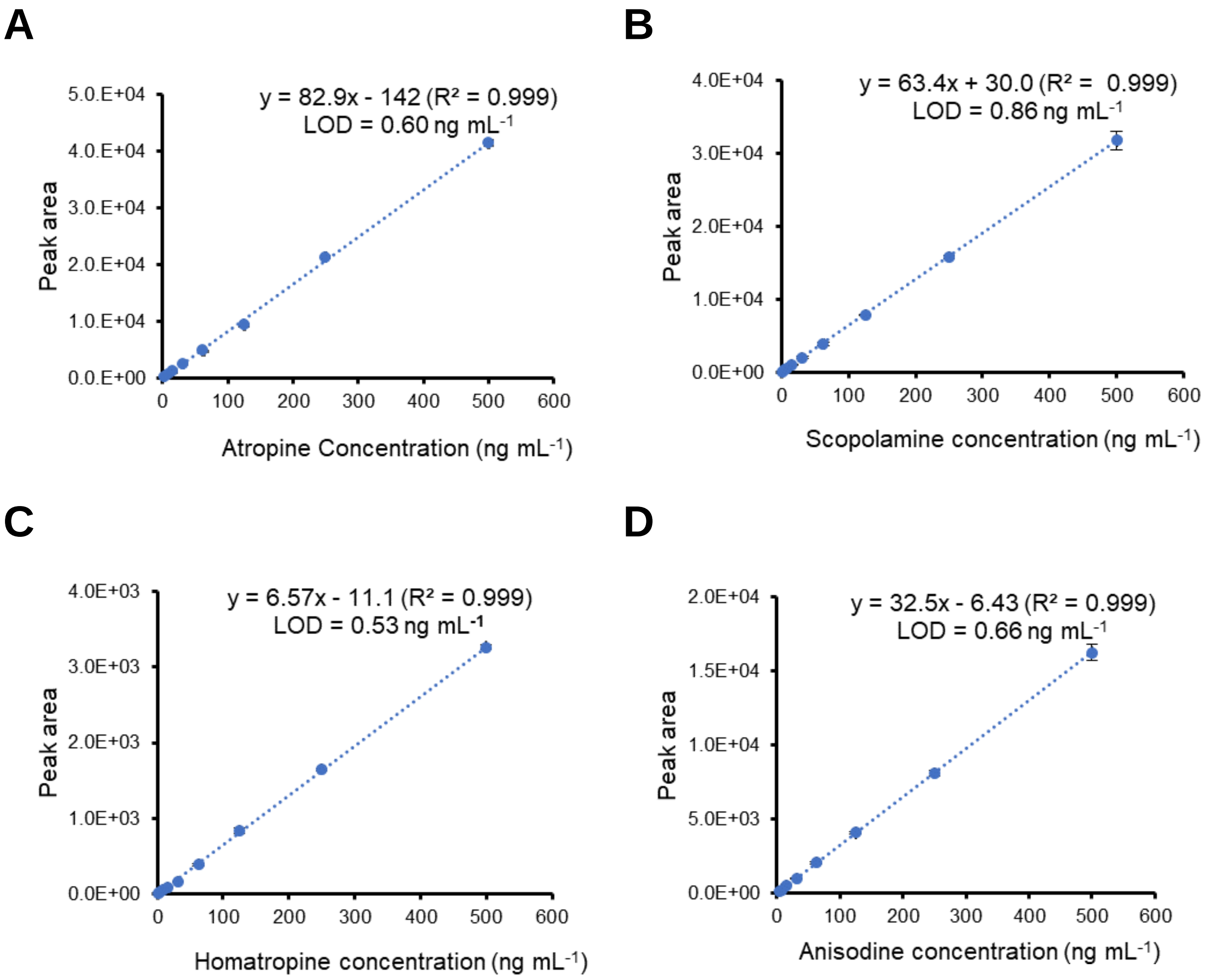

Figure S4. Calibration curves between peak area and concentration of atropine, scopolamine, homatropine, and anisodine constructed by HPLC-MS/MS. Error bars represent the standard deviation (n = 3).

1.00 mg·L^{-1} atropine (ATR), scopolamine (SCO), homatropine (HOM) and anisidine (ANI) stock solutions were diluted with 0.1% (v/v%) formic acid in water to obtain a calibration solution with concentrations of 1.00, 3.91, 7.81, 15.63, 62.50, 125.00, 250.00, 500.00 ng·mL^{-1}. 10 µL of each solution was injected for HPLC-MS/MS analysis in positive mode. The constructed calibration curves were used as external standard to quantify the concentration of ATR, SCO, HOM, and ANI in the samples and extracts.

The limit of detection (LOD) was calculated following:

$$\mathrm{LOD} = 3 \times \frac{\sigma_1}{S} \quad (9)$$

Where S = slope of the calibration curve and σ_1 = standard deviation of the response at 1.00 PPB.

`

Table S2. Prusa Original i3 MK3S+ slicer settings used to print the SLE-filter attachment and indirect competitive lateral flow immunoassay cassette.

**Settings**	
**Layer height**	0.1 mm
**Solid layers top**	9 layers
**Solid layers bottom**	7 layers
**Infill density**	15%
**Infill pattern**	Gyroid
**Filament**	PolyLite PLA Pro
**Perimeter print speed**	35 mm/s
**Supports print speed**	50 mm/s
**Nozzle temperature first layer**	200°C
**Nozzle temperature**	190°C
**Bed temperature**	60°C
**Filament cooling**	100%
**Nozzle diameter**	0.4 mm
**Retraction**	0.4 mm
**Z-lift**	0.2 mm

`

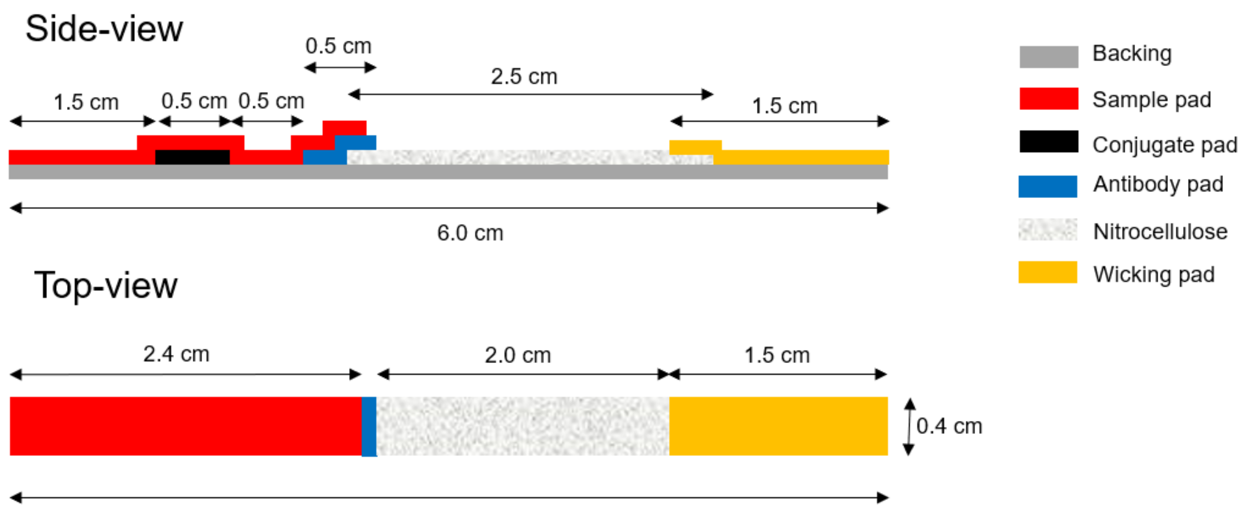

Figure S5. Schematic overview of indirect competitive lateral flow immunoassays design (icLFIA, 4 mm x 60 mm) with conjugate and antibody pad. The icLFIA is constructed on a 4 mm × 60 mm backing card. A sample pad (red, 4 mm × 20 mm), conjugate pad (black, 4 mm × 5 mm), antibody pad (blue, 4 mm × 5 mm), nitrocellulose (NC) membrane (white, 4 mm × 25 mm), and absorbent pad (yellow, 4 mm × 15 mm) has been laminated on top of the backing card (gray, 4 mm × 60 mm). The absorbent pad (yellow) and antibody pad (blue) partially overlay the NC-membrane (white). The sample pad (red) overlays the conjugate pad (black) and antibody pad (blue).

`

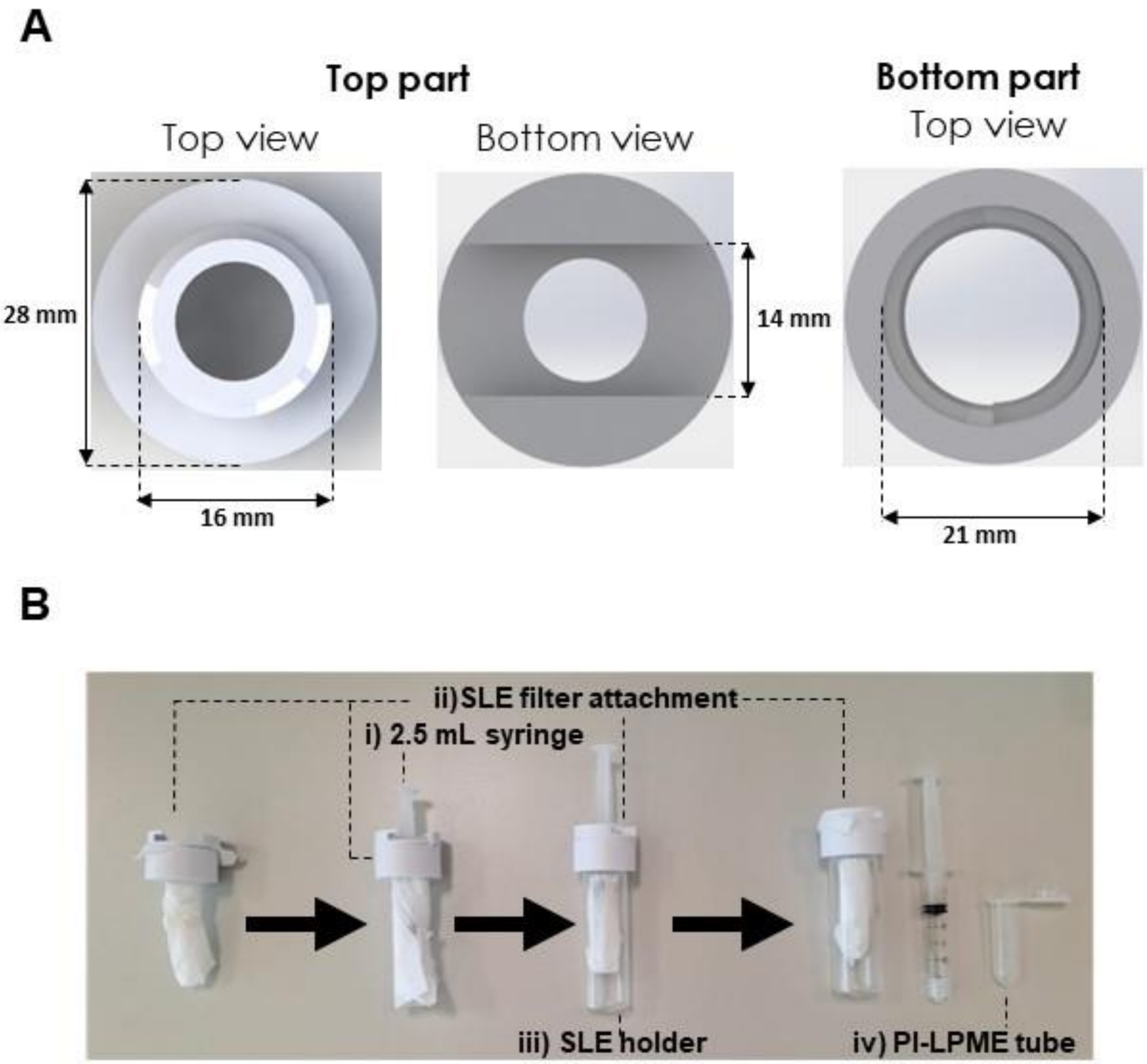

Figure S6. Construction and operation of the solid liquid extraction (SLE) filter attachment. The part numbers correspond to those in Figure 2 of the main text and Protocol S1. **(A)** Computer-aided design (CAD) image and specifications of the top and bottom part of the solid liquid extraction (SLE) filter attachment. The top part of the SLE filter attachment consists of two cylinders with a diameter of 16 mm and 28 mm. The bottom part of the SLE filter attachment consists of one cylinder with a diameter of 28 mm and an internal hole of 21 mm. The bottom part of the SLE filter attachment can be screwed on the top part to fixate a tea-filter which is inserted in between. **(B)** Operation of the SLE filter attachment: Through the top cylinder, a syringe (i) can be inserted and fixed. Then, the SLE-filter attachment (ii) can be attached to the SLE holder (iii) to collect the extract without collecting any solid cereal particles. Finally, the syringe (i) can be released from the SLE-filter attachment (ii) by turning, to allow filling the PI-LPME tube (iv) with SLE-extract for the next step of the workflow.

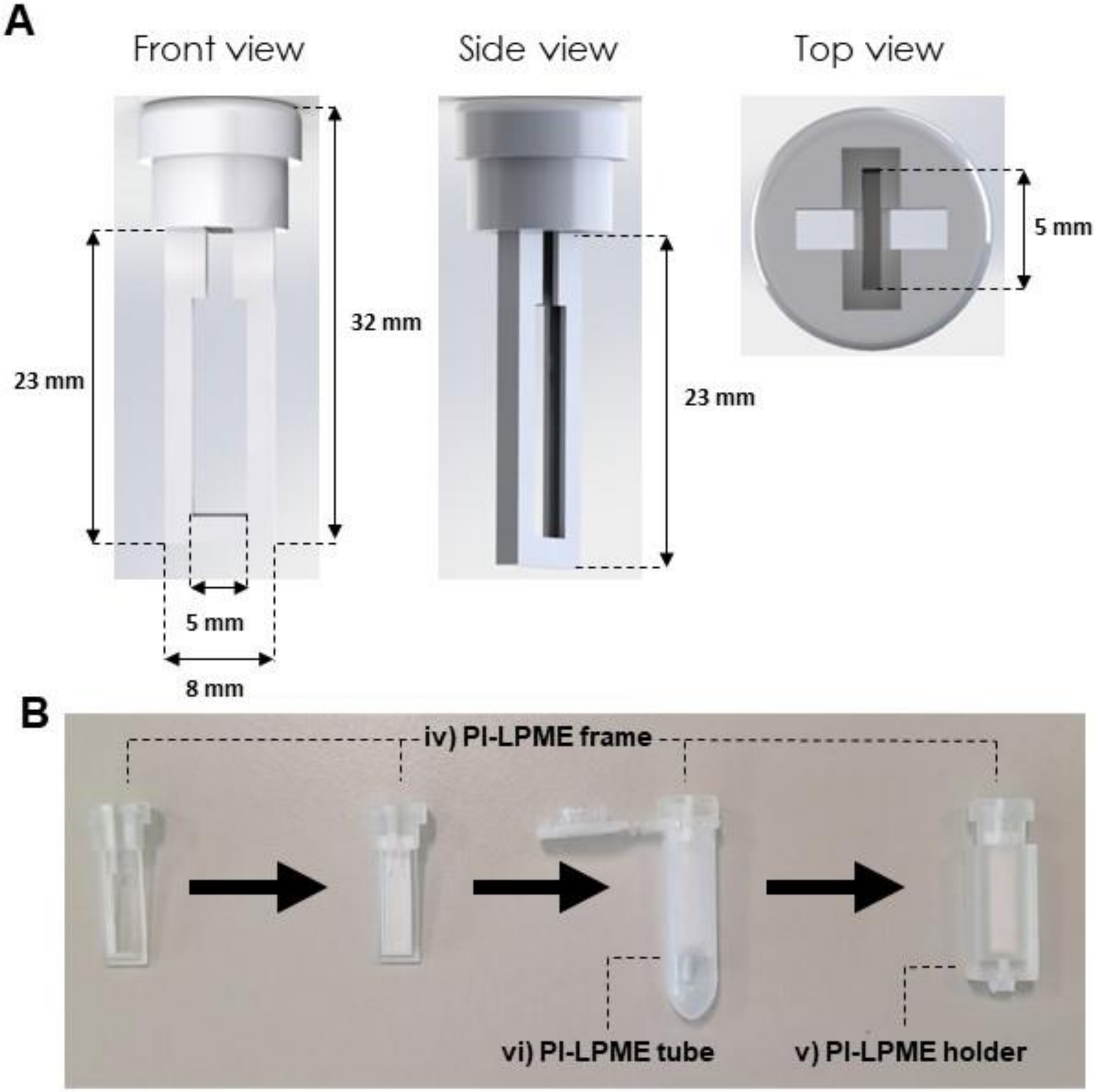

Figure S7. Construction and operation of the 3D-printed paper-immobilized liquid phase microextraction (PI-LPME) frame. The part numbers correspond to those in Figure 2 of the main text and Protocol S1. (**A**) Computer-aided design (CAD) image and specifications of PI-LPME frame. The PI-LPME frame has a rectangular frame (0.9 mm × 6 mm × 23 mm) and the top of the PI-LPME frame consist of two cylinders with a diameter of 10 and 12 mm, to fit as a cap on the 2 mL PILPME tube. (**B**) Operation of the PI-LPME frame: In the PI-LPME frame (iv) a piece of paper (0.3 mm × 5 mm × 23 mm), pre-wetted with 10 µl 0.1% formic acid in water, can be inserted. After this the PI-LPME frame (iv) can be positioned in the PI-LPME tube (vi) to extract the tropane alkaloids from the extract. After the PI-LPME, the PI-LPME frame can be inserted in the PI-LPME holder (v) to elute the tropane alkaloids from the paper.

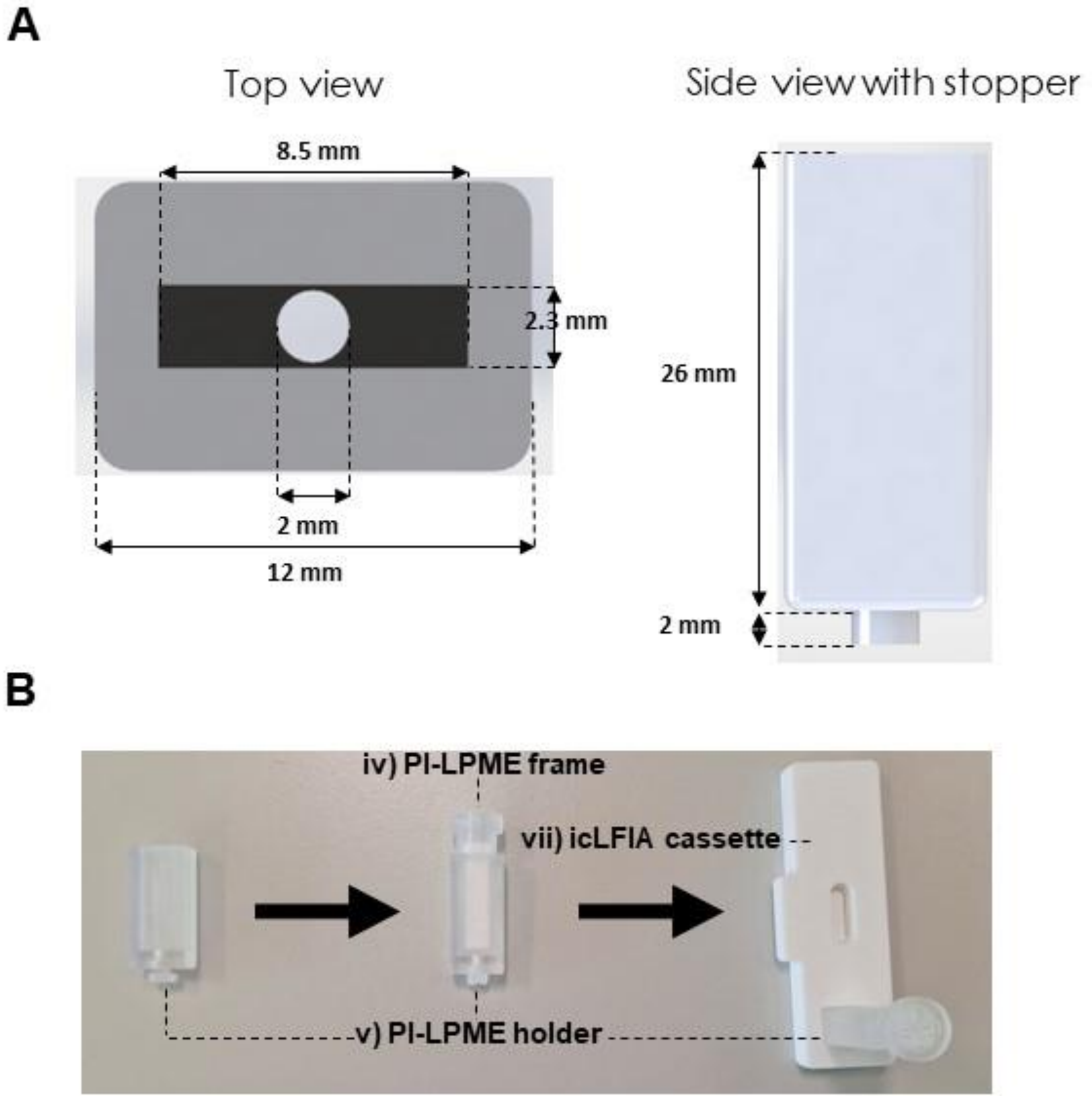

Figure S8. Construction and operation of the 3D-printed paper immobilized liquid phase microextraction (PI-LPME) holder. The part numbers correspond to those in Figure 2 of the main text and Protocol S1. (**A**) Computer-aided design (CAD) image and specifications of the PI-LPME holder (8 mm × 12 mm × 26 mm). The PI-LPME holder has a rectangular gap of (8.5 mm × 2 mm × 23 mm) and a hole (Ø = 2 mm) at the bottom of the PI-LPME holder which can be closed with a stopper. (**B**) Operation of the PI-LPME holder: The PI-LPME holder (v) can be filled with 200 µL of running buffer, after which the PI-LPME frame (iv) can be inserted to elute the tropane alkaloids from the PI-LPME paper. Then, the PI-LPME holder (v) can be unplugged and connected to the icLFIA cassette (vii). The running buffer can be pressed out of the PI-LPME holder (v) by moving the PI-LPME frame (iv) up and down.

`

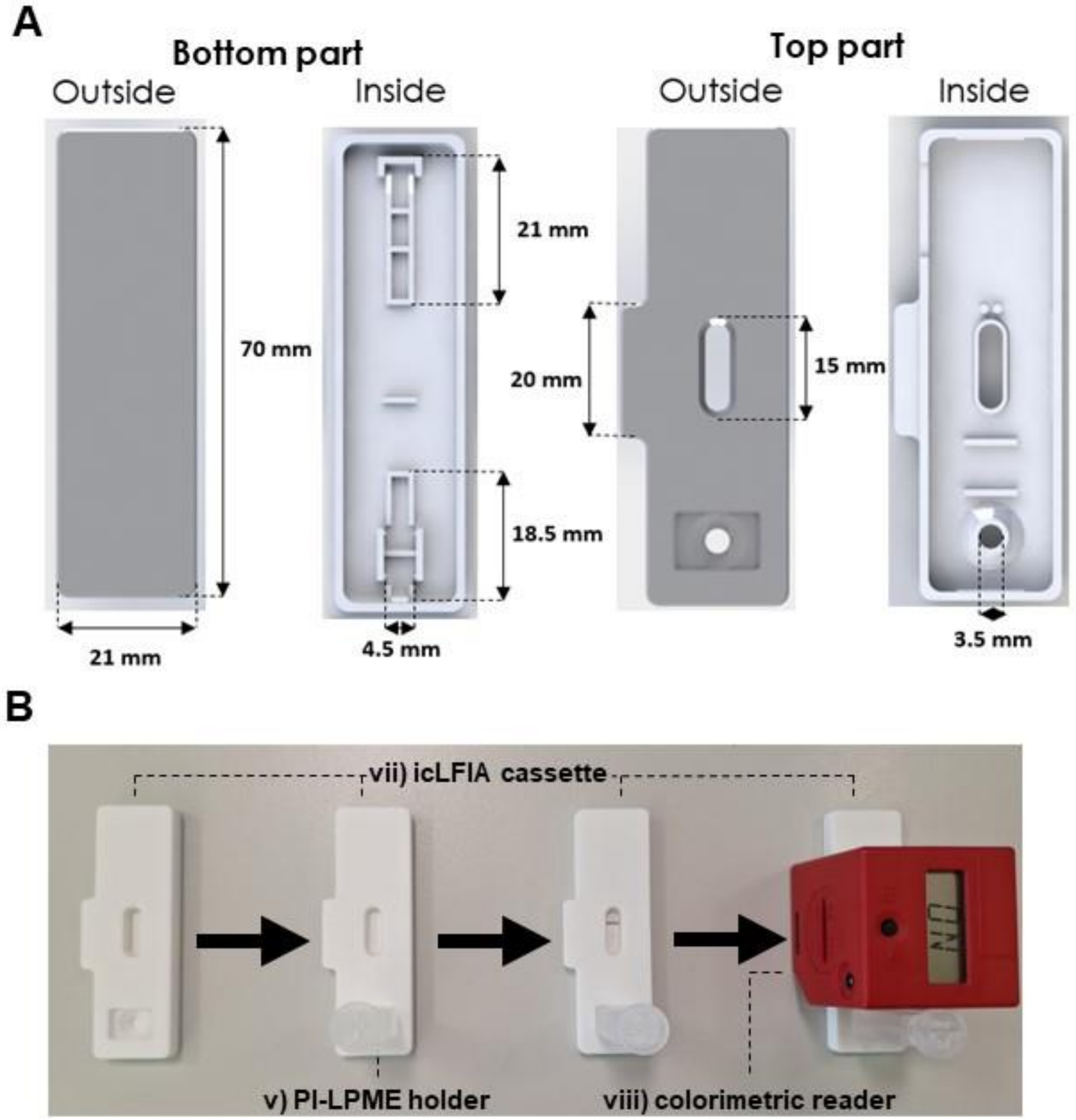

Figure S9. Construction and operation of the 3D-printed indirect competitive lateral flow immunoassay (icLFIA) cassette. The part numbers correspond to those in Figure 2 of the main text and Protocol S1. (**A)** Computer-aided design (CAD) image and specifications of the top and bottom part of the icLFIA cassette (70 mm× 21 mm × 6 mm). Outside and inside of the bottom-part of the icLFIA cassette (70 mm × 21 mm × 2 mm) designed for 60 mm × 4 mm icLFIA strips. Outside and inside of the top-part of the icLFIA cassette (70 mm × 21 mm × 4 mm). (**B**) Operation of the 3D-printed icLFIA cassette: An icLFIA can be inserted in the icLFIA cassette (vii), after this the PI-LPME holder (v) can be connected to the icLFIA cassette and the icLFIA run. Subsequently, a colorimetric reader (viii) can be connected to measure the intensity of the test and control line.

`

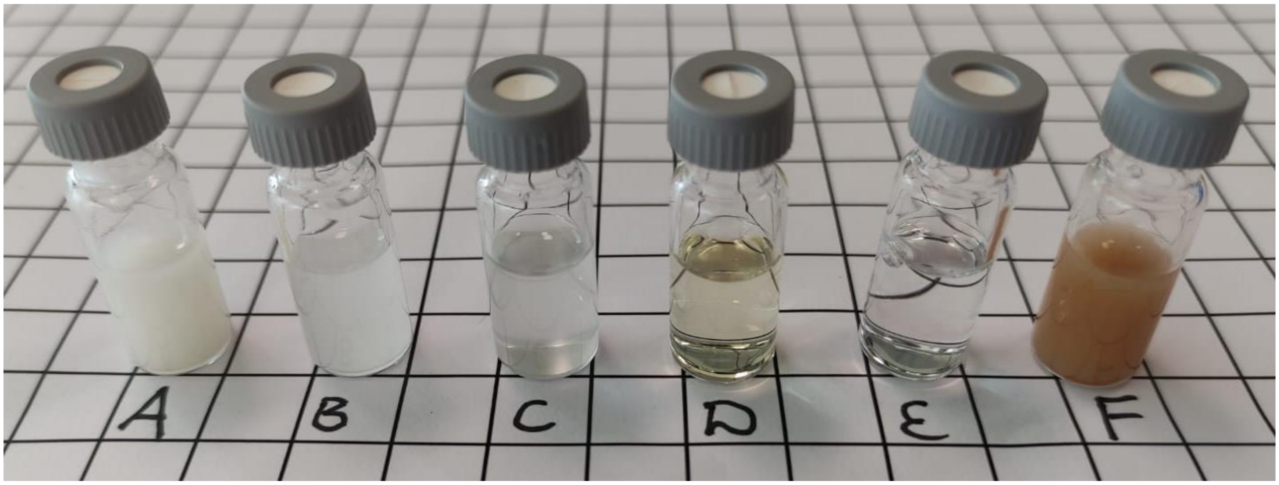

Figure S10. Extract composition with different extraction solvents by extracting 1 gram of buckwheat cereals for 30 min: **(A)** 10 mL 0.1% formic acid in water, **(B)** 10 mL ACN **(C)** 10 mL butyl acetate **(D)** 2 mL 0.05M NaOH in water followed by 10 mL ACN, (**E)** 2 mL 0.05M NaOH in water followed by 10 mL butyl acetate **(F)** 10 mL 0.05 M NaOH in water. From each extract, 1 mL was collected with the SLE collector and placed in an HPLC-vial.

`

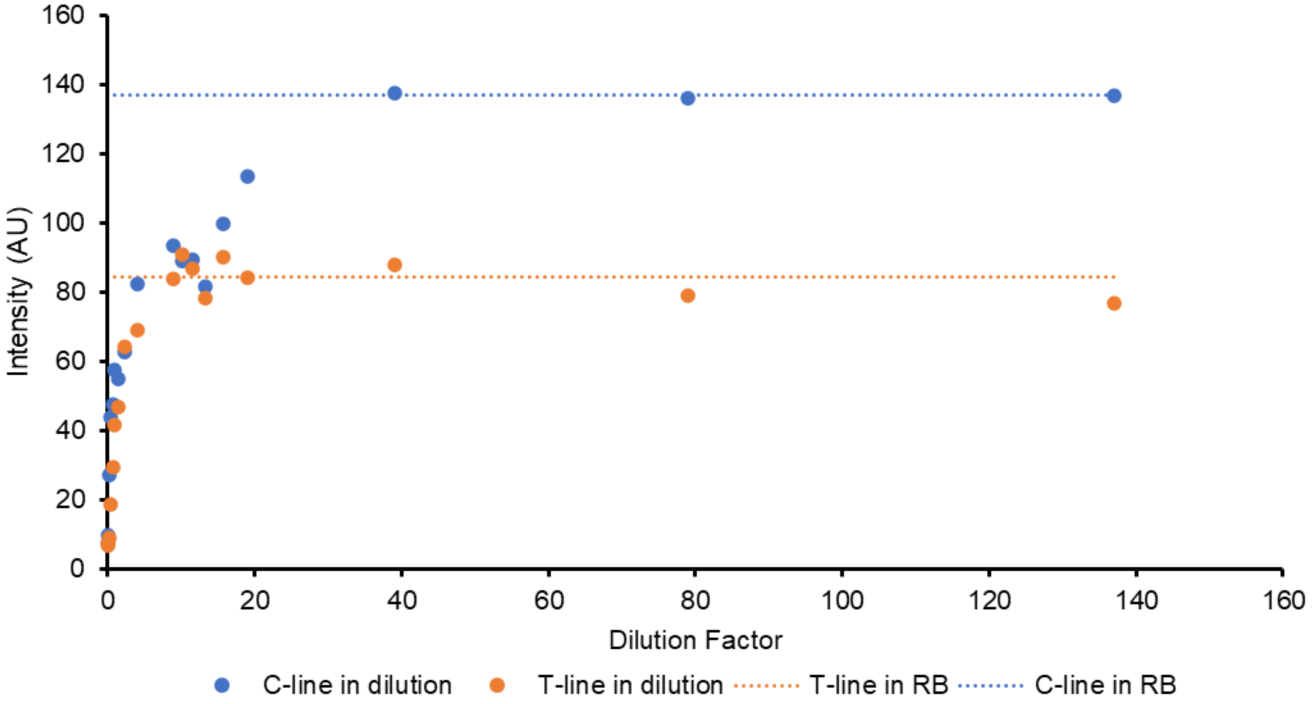

Figure S11. The intensity of the T-line and C-line of the indirect competitive lateral flow immunoassay at different dilutions of blank cereal extract with running buffer (RB, n = 1). The intensity of the T-line and C-line was measured with a digital colorimetric reader. The dilution factor was calculated by dividing the volume of RB added by the volume of extract.

`

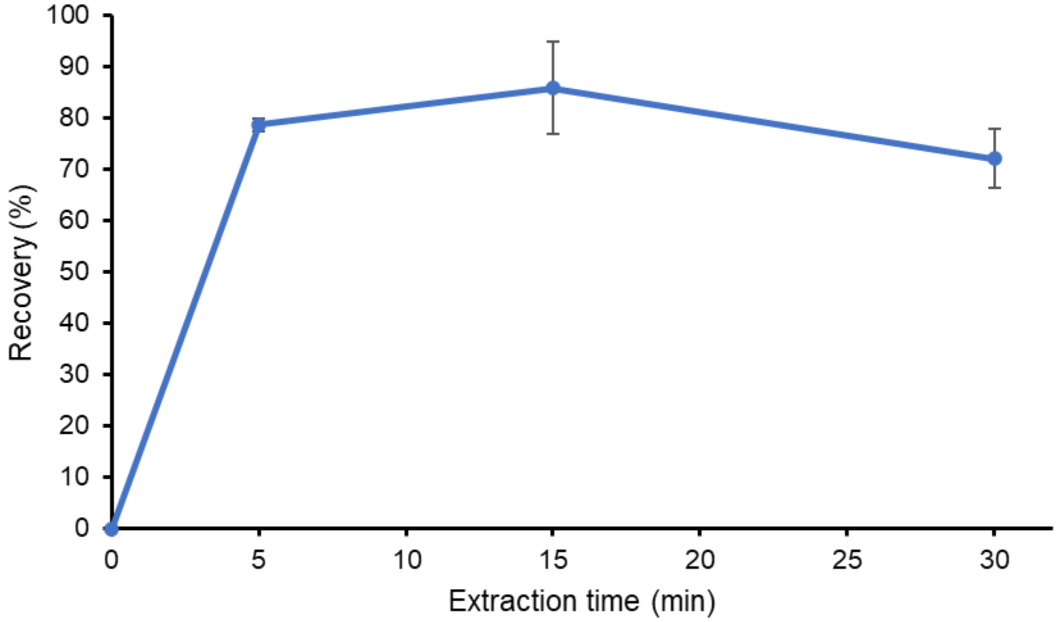

Figure S12. ATR recovery (%) after extracting one gram of cereals spiked with ATR for 5, 15, and 30 min. Dry buckwheat cereals were pre-wetted with 2 mL of 0.05M NaOH in water, and after 20 seconds of manual shaking, extracted with 10 mL butyl acetate. The extracts were collected with the 2.5 mL syringe inserted in the SLE-filter attachment and analyzed by LC-MS/MS. Error bars represent the standard deviation (n = 3).

`

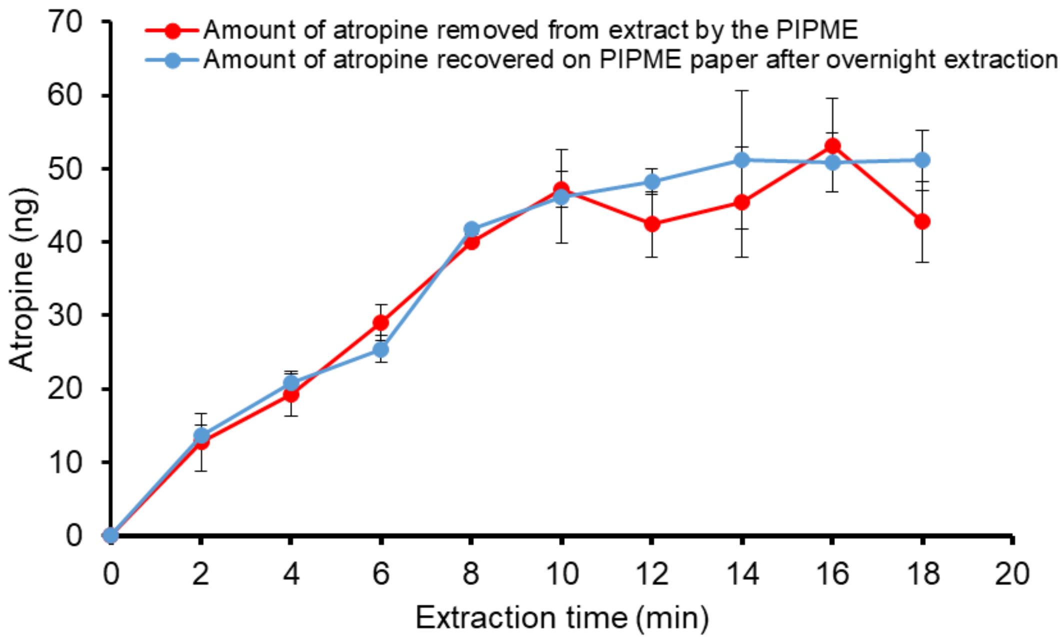

Figure S13. Amount of atropine (ng) removed from the spiked butyl acetate layer (red) and recovered on the pre-wetted paper (5 mm × 23 mm), after an overnight extraction in 200 µL 0.1% (v/v%) formic acid in water (Blue). For the paper immobilized liquid phase microextraction (PILPME), the paper (5 mm × 23 mm) in the PILPME frame (iv) was wetted with 10µL 0.1% (v/v%) FA, and subsequently submerged in the PILPME tube (i) containing 1.8 mL of spiked butyl acetate. For the overnight extraction the paper (5 mm × 23 mm) was transferred to a 1.5 mL Eppendorf tube. The recovery (%) was determined with LC-MS/MS analysis. Error bars represent the standard deviation (n = 3).

`

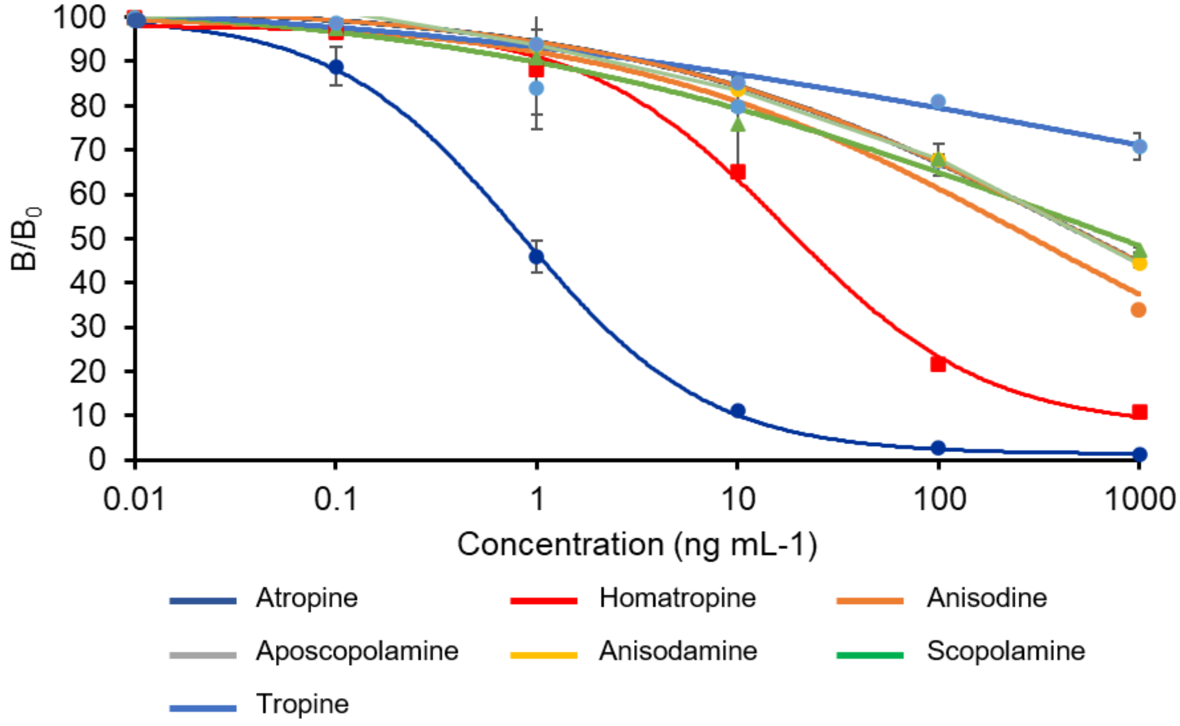

Figure S14. Dose-response curves of the atropine-specific monoclonal antibody combined with the atropine-bovine serum albumin conjugate for atropine (dark blue), homatropine (red), Anisodine (orange), aposcopolamine (grey), anisodamine (yellow), scopolamine (green) and tropine (light blue) in the singleplex indirect competitive microsphere-based immunoassay. Error bars represent the standard deviation (n = 2).

`

Table S3. Sensitivity and specificity of the singleplex indirect competitive microsphere-based immunoassay (icMI) with the hyoscamine specific mAb combined with the ATR-BSA conjugate for atropine, homatropine, aposcopolamine, scopolamine, anisidine, anisodamine, and tropine.

**Alkaloid**	**IC_{50} (ng mL^{-1})**	**Cross-reactivity (%)**
Atropine	0.79	100%
Homatropine	16.75	4.71%
Aposcopolamine	>100	<0.79%
Scopolamine	>100	<0.79%
Anisadine	>100	<0.79%
Anidosamine	>100	<0.79%
Tropine	>100	<0.79%

The IC_{50} was computed by fitting a five parameter logistic curve through the microsphere-based immunoassay results with GraphPad Prism version 10 (Domatics; Boston, USA). The cross-reactivity (CR) was calculated following:

(5) $$CR = \frac{IC_{50}[ATR]}{IC_{50}[competitior]} \times 100\%$$

Where IC_{50}[ATR] = half-maximum inhibitory concentration of ATR (ng mL^{-1}),and IC_{50}[competitor] = half-maximum inhibitory concentration of another TA (ng mL^{-1}).[5]

`

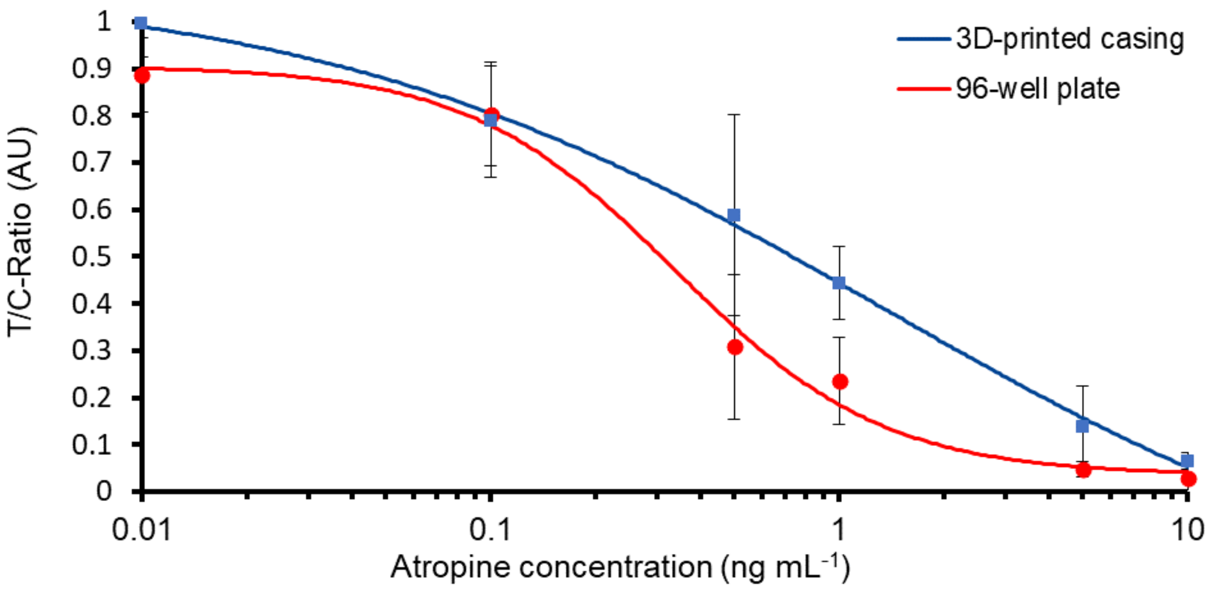

Figure S15. Determined T/C-ratios after indirect competitive lateral flow immunoassay (icLFIA) read-out with a colorimetric reader (viii) by testing atropine dissolved in running buffer at different concentrations. The icLFIAs were run in a 96-well plate (red) or by adding 100 µL of the test solution with atropine to the icLFIA in the 3D-printed cassette (vii, blue). Colorimetric read-out was performed after 10 min. Error bars represent the standard deviation (n=3).

`

AGREEprep

Analytical Greenness Metric for Sample Preparation

08/08/2024 12:14:18

Report

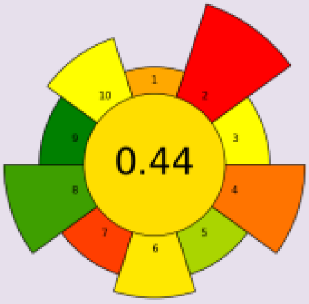

#	Criterion		Score	Weight
1.	Sample preparation placement:	On site	0.33	1
2.	Hazardous materials:	12 [g or mL]	0.00	5
3.	Sustainability, renewability, and reusability of materials:	50-75% of reagents and materials are sustainable or renewable, but can only be used ONCE	0.50	2
4.	Waste:	12 [g or mL]	0.23	4
5.	Size economy of the sample	Mass or volume of the sample: 1 [g or mL]	0.67	2
6.	Sample throughput:	7 [samples/h]	0.46	3
7.	Integration and automation	Sample prep. steps: 4 steps, Manual systems	0.13	2
8.	Energy consumption:	16 [W]	0.88	4
9.	Post-sample preparation configuration for analysis:	Simple, readily available detection: smartphones, desktop scanners, paper strips, etc.	1.00	2
10.	Operator's safety:	2 hazards	0.50	3

Figure S16. Assessment of the Greenness of the sample preparation workflow of the modular workflow using AGREEprep. Each criteria is assigned their default weight as described by Wojnowski *et al.*[11]

`

References

(1) Veršilovskis, A.; Mulder, P. P. J.; Pereboom-de Fauw, D. P. K. H.; de Stoppelaar, J.; de Nijs, M. Simultaneous quantification of ergot and tropane alkaloids in bread in the netherlands by LC-MS/MS. Food Addit. Contam. Part B Surveill. 2020, 13 (3), 215–223. https://doi.org/10.1080/19393210.2020.1771777.

(2) Jakabová, S.; Vincze, L.; Farkas, Á.; Kilár, F.; Boros, B.; Felinger, A. Determination of tropane alkaloids atropine and scopolamine by liquid chromatography-mass spectrometry in plant organs of datura species. J. Chromatogr. A. 2012, 1232, 295–301. https://doi.org/10.1016/j.chroma.2012.02.036.

(3) Zou, R.; Guo, Y.; Chen, Y.; Zhao, Y.; Zhao, L.; Zhu, G.; Liu, Y.; Peters, J.; Guo, Y. Profiling of a unique broad-specific antibody and its application to an ultrasensitive fluoroimmunoassay for five N-methyl carbamate pesticides. *J. Hazard. Mater*. 2022, 426. https://doi.org/10.1016/j.jhazmat.2021.127845.

(4) Angeloni, S.; Cordes, R.; Dunebar, S. XMAP Cookbook: A Collection of Methods and Protocols for developing multiplex assays with Xmap technology; Austin, 2016.

(5) Wang, Z.; Zheng, P.; Wang, J.; He, S.; Ren, Z.; Zhang, Y.; Xiong, J.; Jiang, H. Indirect competitive enzyme-linked immunosorbent assay based on a broad-spectrum monoclonal antibody for tropane alkaloids detection in pig urine, pork and cereal flours. Food. Chem. 2021, 337, 1-8 https://doi.org/10.1016/j.foodchem.2020.127617

(6) Ross, G. M. S.; Salentijn, G. I.; Nielen, M. W. F. A critical comparison between flow-through and lateral flow immunoassay formats for visual and smartphone-based multiplex allergen detection. *Biosensors* 2019, 9 (4). https://doi.org/10.3390/bios9040143.

(7) Mahmoudi, T.; de la Guardia, M.; Baradaran, B. Lateral flow assays towards Point-of-Care cancer detection: a review of current progress and future trends. TrAC, 2020, 125, 1-20 https://doi.org/10.1016/j.trac.2020.115842

(8) Pan, X.; Wang, H.; Li, C.; Zhang, J. Z. H.; Ji, C. *J. Chem. Inf. Model.* **2021,** 61 (7), 3159–3165. https://doi.org/10.1021/acs.jcim.1c00075

(9) Tetko, I. V.; Gasteiger, J.; Todeschini, R.; Mauri, A.; Livingstone, D.; Ertl, P.; Palyulin, V. A.; Radchenko, E. V.; Zefirov, N. S.; Makarenko, A. S.; Tanchuk, V. Y.; Prokopenko, V. V. J. Virtual Computational Chemistry Laboratory - Design and Description. *Comput. Aided. Mol. Des.* **2005,** 19 (6), 453–463. https://doi.org/10.1007/s10822-005-8694-y.

(10) Tetko, I. V.; Tanchuk, V. Y. J. Application of associative neural networks for prediction of lipophilicity in ALOGPS 2.1 Program. *Chem. Inf. Comput.* Sci. **2002,** 42 (5), 1136–1145. https://doi.org/10.1021/ci025515j.

(11) Wojnowski, W.; Tobiszewski, M.; Pena-Pereira, F.; Psillakis, E. AGREEprep – analytical greenness metric for sample preparation. *TrAC*, 2022, 149, 1-9 https://doi.org/10.1016/j.trac.2022.116553
